# Functional Analysis of PGRP-LA in *Drosophila* Immunity

**DOI:** 10.1371/journal.pone.0069742

**Published:** 2013-07-26

**Authors:** Mathilde Gendrin, Anna Zaidman-Rémy, Nichole A. Broderick, Juan Paredes, Mickaël Poidevin, Alain Roussel, Bruno Lemaitre

**Affiliations:** 1 Global Health Institute, School of Life Sciences, Ecole Polytechnique Fédérale Lausanne (EPFL), Lausanne, Switzerland; 2 Centre de Génétique Moléculaire, CNRS/Université Pierre et Marie Curie, Gif-sur-Yvette, France; 3 Architecture et Fonction des Macromolécules Biologiques, CNRS/Aix Marseille Université, Marseille, France; Ecole Normale Supérieur de Lyon, France

## Abstract

PeptidoGlycan Recognition Proteins (PGRPs) are key regulators of the insect innate antibacterial response. Even if they have been intensively studied, some of them have yet unknown functions. Here, we present a functional analysis of PGRP-LA, an as yet uncharacterized *Drosophila* PGRP. The *PGRP-LA* gene is located in cluster with *PGRP-LC* and *PGRP-LF*, which encode a receptor and a negative regulator of the Imd pathway, respectively. Structure predictions indicate that PGRP-LA would not bind to peptidoglycan, pointing to a regulatory role of this PGRP. *PGRP-LA* expression was enriched in barrier epithelia, but low in the fat body. Use of a newly generated *PGRP-LA* deficient mutant indicates that *PGRP-LA* is not required for the production of antimicrobial peptides by the fat body in response to a systemic infection. Focusing on the respiratory tract, where *PGRP-LA* is strongly expressed, we conducted a genome-wide microarray analysis of the tracheal immune response of wild-type, *Relish*, and *PGRP-LA* mutant larvae. Comparing our data to previous microarray studies, we report that a majority of genes regulated in the trachea upon infection differ from those induced in the gut or the fat body. Importantly, antimicrobial peptide gene expression was reduced in the tracheae of larvae and in the adult gut of *PGRP-LA*-deficient *Drosophila* upon oral bacterial infection. Together, our results suggest that PGRP-LA positively regulates the Imd pathway in barrier epithelia.

## Introduction

*Drosophila*, in contrast to mammals, lacks adaptive immunity and therefore relies entirely on innate immunity for defense against invading pathogens [1], [2]. Microorganisms are recognized through the interaction between microbial compounds and host pattern-recognition receptors. In insects, the peptidoglycan recognition proteins (PGRPs) are a major class of pattern-recognition receptors that sense bacteria by interacting with peptidoglycan and regulate host antibacterial defenses. In *Drosophila*, the Toll and Imd pathways are the two major signaling cascades regulating the massive expression of antimicrobial peptide genes and other immune genes by the fat body following a systemic infection [3]–[5]. The Toll pathway is strongly induced by Gram-positive bacteria and fungi, and controls the expression of several genes, notably the antifungal peptide gene *Drosomycin*; the Imd pathway is strongly induced by Gram-negative and bacillus-shaped Gram-positive bacteria and regulates the expression of genes such as *Diptericin*, encoding an antibacterial peptide [6]. Activation of both pathways by bacteria is achieved through the sensing of specific forms of peptidoglycan by PGRPs. Peptidoglycan is an essential cell wall component of bacteria, composed of long glycan chains with alternating N-acetylglucosamine and N-acetylmuramic acid residues that are cross-linked to each other by short peptide bridges. The third residue of these stem peptides differs between bacteria: it is a lysine in Gram-positive cocci and a meso-diaminopimelic acid (DAP) in both Gram-negative bacteria and Gram-positive bacilli, such as *Bacillus* and *Listeria* species [7]. Studies using highly purified bacterial compounds have shown that the highest Toll pathway activity is observed upon injection of Lysine-type peptidoglycan, while the Imd pathway is activated by DAP-type peptidoglycan [8]. Further studies have shown that both polymeric and monomeric DAP-type peptidoglycan can activate the Imd pathway. A specific monomer, the GlcNAc-MurNAc(anhydro)-L-Ala-γ-D-Glu-*meso*-DAP-D-Ala, also known as tracheal cytotoxin (TCT), has been identified as the minimal peptidoglycan motif capable of efficient induction of the Imd pathway [9], [10].

PGRPs form a conserved family of proteins sharing a 160 amino acid domain (the PGRP domain) with similarities to bacteriophage T7 lysozyme, a zinc-dependent N-acetylmuramoyl-L-alanine amidase that removes peptides from the glycan chains of peptidoglycan [11], [12]. The *Drosophila* genome encodes 13 PGRPs, some of which retain amidase properties. The PGRPs of this subgroup, referred to as catalytic PGRPs, have demonstrated (PGRP-SC1A/B, LB, SB1) or predicted (PGRP-SB2, SC2) zinc-dependent amidase activity, which reduces or eliminates the ability of peptidoglycan to elicit an immune response [13]–[15]. PGRP-LB and to a lesser extent PGRP-SC1A/SC1B/SC2 have been shown to down-regulate the Imd pathway activity by scavenging peptidoglycan [16]–[18]. The exact function of PGRP-SB1/SB2 is not yet clear: it was proposed that this secreted PGRP could function as an antibacterial protein [15], but a recent genetic analysis did not identify any immune phenotype [19]. The non-catalytic PGRPs (PGRP-SA, SD, LA, LC, LD, LE, LF) lack the zinc-binding residues required for amidase activity but some of them retain the ability to bind peptidoglycan and function as bacteria sensors. PGRP-SA and PGRP-SD are secreted proteins circulating in the hemolymph that have been shown to activate the Toll pathway in response to the Lysine-type peptidoglycan found in most Gram-positive bacteria [20], [21]. The receptor PGRP-LC, located at the plasma membrane, induces the Imd pathway when activated by DAP-type peptidoglycan [22]–[24]. PGRP-LE is produced in both extracellular and intracellular forms and has been shown to participate in the sensing of bacteria containing DAP-type peptidoglycan in two different manners. A secreted fragment of PGRP-LE corresponding to the PGRP domain alone enhances PGRP-LC-mediated peptidoglycan recognition on the cell surface [25]. In contrast, the full-length form of PGRP-LE is cytoplasmic and acts as an intracellular receptor for monomeric peptidoglycan, effectively bypassing the requirement for PGRP-LC [26]. While PGRP-LC is the main receptor upstream of the Imd pathway in the fat body, both PGRP-LC and PGRP-LE account for the sensing of Gram-negative bacteria upstream of the Imd pathway in the gut [27], [28]. A gene in cluster with *PGRP-LC*, *PGRP-LF* encodes a transmembrane protein with two PGRP domains. Studies have indicated that PGRP-LF does not bind peptidoglycan but inhibits the activation of PGRP-LC by competing with PGRP-LC dimerization [29], [30]. The functions of PGRP-LD and PGRP-LA are not yet known.

In this study, we report a functional analysis of PGRP-LA, a non-catalytic PGRP encoded by a gene of the *PGRP-LC* genomic cluster. *PGRP-LA* expression is enriched in several barrier epithelia such as the hindgut and tracheae whereas its expression in the fat body is low [31]. Based on over-expression, deletion and rescue experiments, this work suggests that *PGRP-LA* has a regulatory role and is involved in the fine-tuning of the Imd pathway in barrier epithelia. Our study also includes a genome-wide analysis of gene expression in tracheae in the presence or absence of *PGRP-LA* and *Relish*. Comparing this analysis with previous studies monitoring the fat body and gut responses to bacterial infection reveals a high tissue-specificity of the pool of genes regulated upon infection.

## Results

### Structure predictions indicate that PGRP-LA would not bind to peptidoglycan

**Figure 1:**
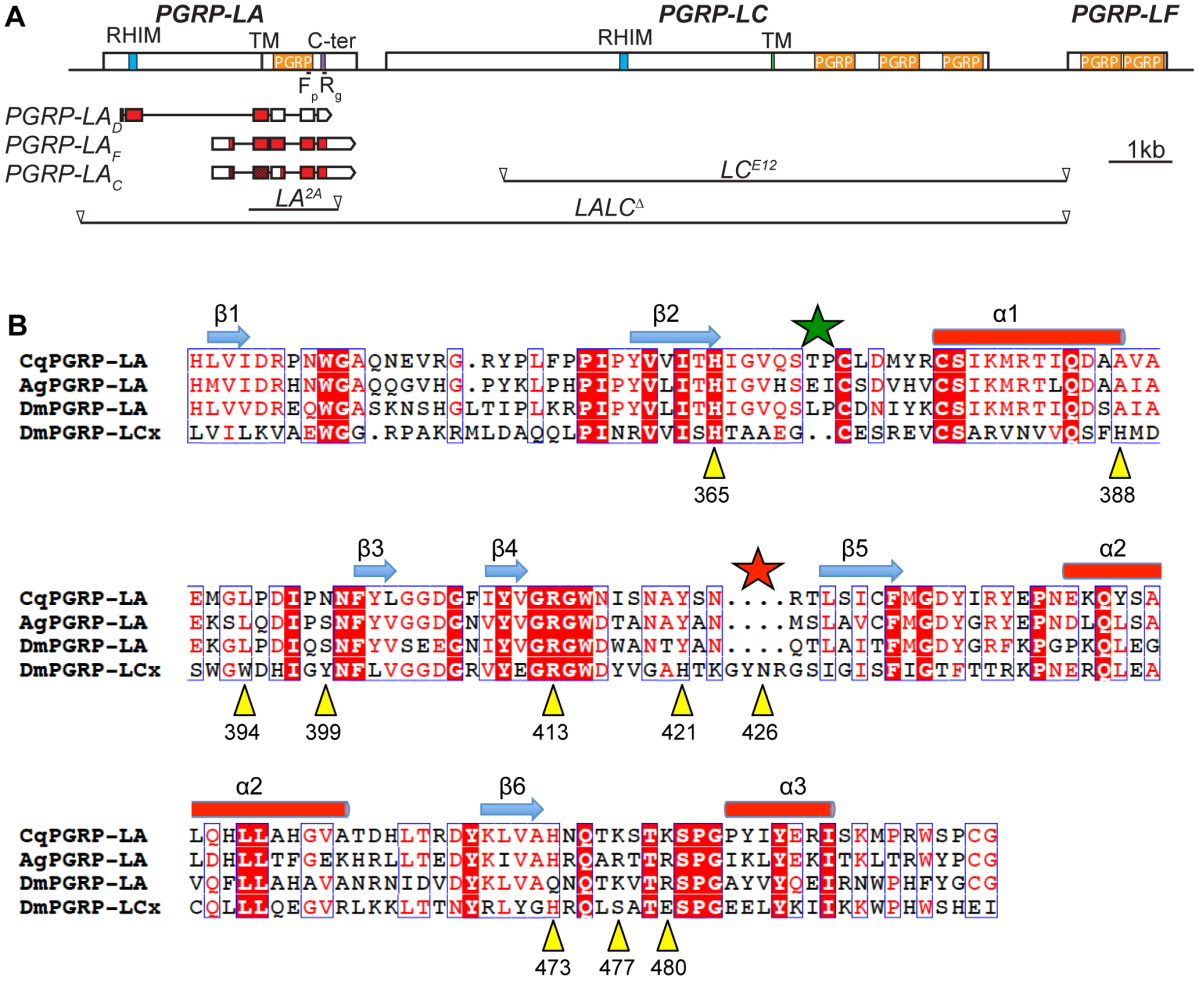
Description of *PGRP-LA* genomic locus and isoforms. **A.** Scheme of the locus containing *PGRP-LA*, *PGRP-LC* and *PGRP-LF*. Each gene contains at least one PGRP domain (orange) and PGRP-LA and LC contain a transmembrane domain (TM, green) and a RHIM motif (blue). No signal peptide has been predicted in the *PGRP-LA* sequence, and the C-terminal sequence (purple) contains 2 Cys residues. *PGRP-LA* encodes three isoforms, depicted under the gene: boxes represent the exons, of which the coding sequence is colored in red. *PGRP-LA^2A^* (*LA^2A^*) deletion was performed by imprecise excision of the *P*-element G14937 (KAIST library) and *PGRP-[LA,LC]^Δ^ (LALC^Δ^)* by FRT mediated deletion of the region between the P-elements 1930 and 4396. *PGRP-LC^E12^* (*LC^E12^*) deletion has already been published [22]. In PGRP-LA_C_, the hatched box represents a sequence between a start and a stop codon, but which is not predicted to be the coding sequence (Flybase). Fp, Rg: localization of the primers used for RT-qPCR. B. Alignment of the proteic sequences of the PGRP domains of PGRP-LA in *Drosophila* (*Dm*), *Anopheles gambiae* (*Ag*) and *Culex quinquefasciatus* (*Cq*) and of *Drosophila* PGRP-LCx, of which the crystal structure has already been solved. Blue boxes contain conserved amino acids (identities and similarities are highlighted and written in red respectively). The residues that are directly in contact with TCT in the structure of the complex with PGRP-LCx [37] are marked with yellow triangles. The numbering corresponds to PGRP-LCx. The two residues insertion in the β2-α1 loop and the four residues deletion in the β4–β5 loop are denoted with green and red stars, respectively.

*PGRP-LA* is located at the 5′ boundary of a cluster of three genes that includes *PGRP-LC* and *PGRP-LF*. It encodes three isoforms, which are referred to here as PGRP-LA_D_, LA_F_ and LA_C_ (**Figure 1A**), following Flybase nomenclature, but which were previously referred to as PGRP-LAa, LAb and LAc respectively [32]. Sequence analysis predicted that the isoforms encoded by *PGRP-LA* differ considerably in their protein domain organizations. PGRP-LA_D_ encodes a putative transmembrane protein with an intracellular domain containing a RIP Homotypic Interaction Motif (RHIM) [26], [33], but lacking the PGRP domain. The RHIM domain is also found in PGRP-LC and PGRP-LE and has been shown to be necessary in these receptors for induction of the Imd pathway [26]. PGRP-LA_F_ contains both a putative transmembrane domain and a PGRP domain, a structure similar to that of the PGRP-LC receptor, except its lack of a RHIM domain. PGRP-LA_C_ encodes a short protein of 138 amino acids composed exclusively of a N-terminus-truncated PGRP domain: although the typical PGRP domain structure comprises a central β-sheet composed of six β-strands surrounded by three α-helices, PGRP-LA_C_ lacks the β1 and β2 sheets and a part of the α1 helix (see **Figure 1B**).

The PGRP domain of most PGRPs has been shown to interact with peptidoglycan. Nevertheless, biochemical studies have shown that some PGRPs, namely PGRP-LF and PGRP-LCa, have lost the capacity to bind peptidoglycan and function as a negative regulator and co-receptor of PGRP-LCx, respectively [30], [34]. To get an insight on PGRP-LA function, we analyzed the sequence of its PGRP domain and its conservation among species. *PGRP-LA* is found in several insect species and its sequence is well conserved across species; the *Drosophila* PGRP-LA domain shares 60% identity with *Aedes* and *Culex* and 52% with *Anopheles* (**Figure 1B**). In addition, the PGRP-LA domain sequence shares only 35% identity with PGRP-LE (60/168), 32% with PGRP-LCx (46/142), and 31% with PGRP-LF (52/164). These percentages are lower than the identity rate among other PGRPs (e.g. PGRP-LCx shares 40 to 52% with PGRP-LF, SD, SC1 and SA), but are above the 30% threshold necessary to predict that the folding of PGRP-LA is similar to the folding of other PGRPs [35]. Study of the putative peptidoglycan binding site of PGRP-LA using both the 3D model obtained with the Phyre software [36] and the sequence alignment with PGRP-LCx leads to three main observations. First, among the 10 residues of PGRP-LCx implicated in the binding to TCT [37], only two are conserved in PGRP-LA (**Figure 1B**), although these residues are highly conserved in PGRPs [14], [38]. In particular, His388, which binds to GlcNAc, is replaced by an alanine, Tyr399, which is located in the central part of the binding crevice, is replaced by a serine, and Trp394, which stacks against the elongated side chain of DAP, is replaced by a leucine. In addition several residues, which are not directly in contact with TCT, but are engaged in shaping the binding crevice, are also not conserved in PGRP-LA. This is the case for Thr366, which is replaced by an isoleucine. Second, the PGRP domain of PGRP-LA displays a deletion of four amino acids in the β4–β5 loop (**Figure 1B**), which is known to be crucial for the binding to peptidoglycan, as an insertion of two residues in this loop prevents the binding to peptidoglycan in PGRP-LCa [34]. Third, an insertion of two residues occurs in the β2-α1 loop (**Figure 1B**), which has been shown to stabilize the pyranose ring of the MurNAc sugar of TCT. Considering these three points, it seems very unlikely that PGRP-LA binds peptidoglycan, suggesting that this PGRP is not a receptor but could have a regulatory role.

### *PGRP-LA* is expressed in barrier epithelia and is up-regulated in response to infection

**Figure 2:**
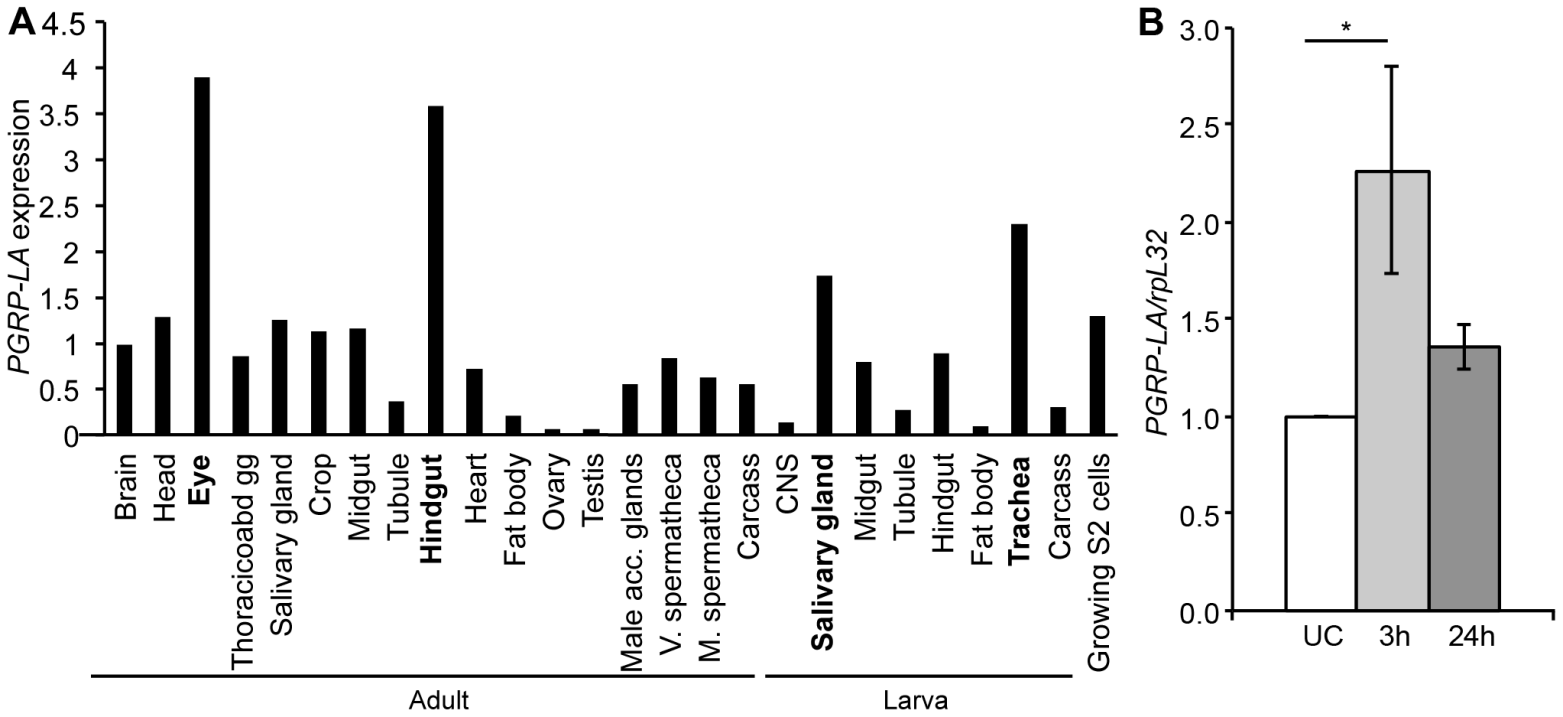
Analysis of *PGRP-LA* expression. **A.** Microarray tissue-specific expression of *PGRP-LA*, data from Flyatlas [31]. Expression is shown as a ratio of *PGRP-LA* mRNA enrichment in each tissue to the average of *PGRP-LA* mRNA enrichment in all the tissues. All 3 isoforms are detected by the *PGRP-LA* probes. Thoracicoabd gg – thoracicoabdominal ganglion, V. and M. spermatheca – virgin and mated spermatheca. **B.** RT-qPCR analysis of *PGRP-LA* expression in whole adults subjected to a septic injury with *Erwinia carotovora carotovora 15 (Ecc15)*. UC – Unchallenged. Data are the mean of 7 repeats, indicated as fold change of UC, and error bars show standard error. Data were analyzed by ANOVA1 followed by Dunnett's multiple comparison test using UC as reference (UC vs 24 h are not significantly different).

*PGRP-LA* was shown to be expressed at a moderate level during most developmental stages and its level of expression is higher in late larvae and prepupae [32], [39]. We confirmed these results by RT-qPCR analysis (**Figure S1A**). Data from FlyAtlas reveal a strong expression of *PGRP-LA* in barrier epithelia, especially in salivary glands and tracheae of larvae and in the hindgut and eyes of adults, while it was weakly expressed in the fat body (9% and 21% of the average expression respectively for larvae and adults, **Figure 2A**) [31]. RT-qPCR with PGRP-LA_D_ or PGRP-LA_C/F_ specific primers shows a similar distribution of these isoforms in all the tissues tested except in Malpighian tubules where PGRP-LA_C_ and _F_ were absent (**Figure S1A**). Previous studies have shown that *PGRP-LA* expression is induced about two-fold in adults upon septic injury [3], [30]. Upon oral infection with *Ecc15*, *PGRP-LA* was also shown to be induced 1.6-fold and 10-fold in adult gut and larval tracheae, respectively [40], [41]. Using RT-qPCR, we confirmed that *PGRP-LA* expression is induced in whole flies after septic injury and in the midgut after oral infection with the Gram-negative bacterium *Erwinia carotovora carotovora 15* (*Ecc15*) (**Figures 2B** **, S1B**). Together, these data indicate that *PGRP-LA* is induced after epithelial and septic infection.

### Over-expression of *PGRP-LA_D_* induces the Imd pathway

**Figure 3:**
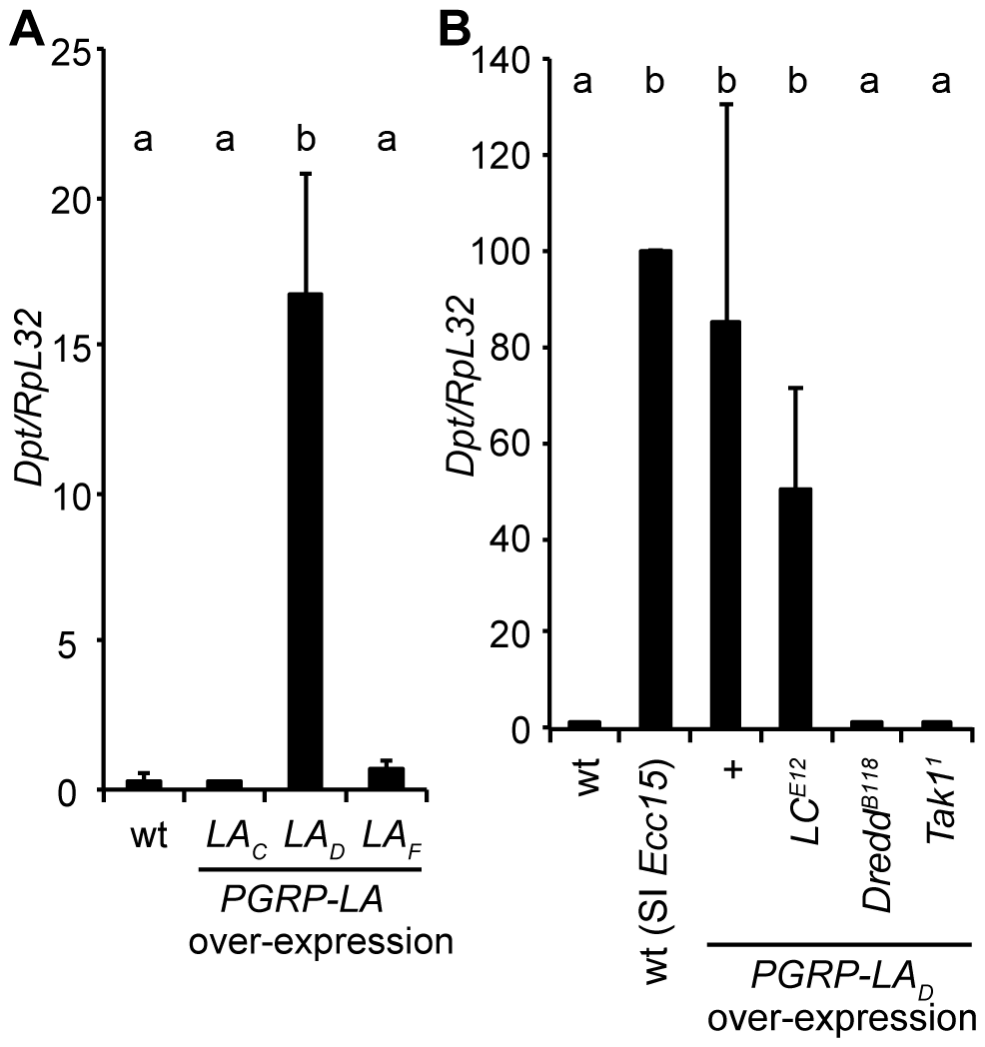
*PGRP-LA_D_* over-expression leads to induction of the Imd pathway. **A.** Measurement of *Diptericin* (*Dpt*) by RT-qPCR in whole males over-expressing each isoform of *PGRP-LA* under the control of the ubiquitous *da-Gal4* driver, using *UAS-PGRP-LA_C_* (*LA_C_*), *UAS-PGRP-LA_D_* (*LA_D_*) and *UAS-PGRPLA_F_* (*LA_F_*) transgenes. **B.** Measurement of *Dpt* by RT-qPCR in *PGRP-LC*, *Dredd*, or *Tak1*-deficient whole males over-expressing *PGRP-LA_D_* under the control of the ubiquitous *da-Gal4* driver. Results are shown as fold change of *Dpt* expression versus wild-type (+) unchallenged controls. Data are expressed as a percentage of *Dpt/RpL32* 6 h after septic injury (SI) and are the mean of three experiments; error bars indicate standard errors. In **A**, **B**, data were analyzed by ANOVA1 followed by Dunnett's multiple comparison test using wt (i.e. da-Gal4 x w) (**A**) and wt (SI) (**B**) as references (a and b groups are statistically different, p<0.01 (**A**) and p<0.05 (**B**)).

Over-expression of PGRP-LE and PGRP-LC is sufficient to activate the Imd pathway, in agreement with their function upstream of this signaling cascade [22], [42]. This prompted us to investigate the effect of the over-expression of PGRP-LA isoforms on the Imd pathway activation. **Figure 3A** shows that, using the *da-Gal4* driver, over-expression in unchallenged flies of PGRP-LA_D_ but not that of PGRP-LA_F_ or PGRP-LA_C_ was sufficient to induce a very high expression level of *Diptericin*, an antibacterial peptide gene used as a read-out of the Imd pathway. *Diptericin* induction by PGRP-LA_D_ required Dredd and Tak1, but not PGRP-LC (**Figure 3B**). We also observed that ubiquitous over-expression of *PGRP-LA_D_* with the *da-Gal4* driver induces some lethality, as observed upon *PGRP-LC* ubiquitous over-expression (data not shown). The observation that PGRP-LA_D_ can activate the Imd pathway, the presence of a RHIM domain, and the location of *PGRP-LA* in the same cluster as PGRP-*LC* and *LF* are suggestive of a role of PGRP-LA in the regulation of the Imd pathway.

### PGRP-LA is dispensable for the induction of a systemic immune response

In order to investigate the role of *PGRP-LA in vivo*, we generated a *Drosophila* strain deficient for *PGRP-LA* by imprecise excision of the *P*-element *G14937* (from the Korea Advanced Institute of Science and Technology library). *PGRP-LA^2A^* mutant bears a deletion of 1401 bp upstream of the *P*-element insertion site, uncovering the PGRP domain sequence. This deletion includes the exons encoding the whole of the PGRP-LA_C_ and LA_F_ isoforms and the last four exons of the PGRP-LA_D_ isoform (**Figure 1A**). In agreement with the molecular characterization, we found that *PGRP-LA^2A^* adults did not express *PGRP-LA* mRNA (see below). In addition, *PGRP-LA^2A^* mutants were viable and fertile and did not show any apparent developmental defects as observed for all the other PGRP deficient lines described so far. We introgressed the *PGRP-LA^2A^* mutation into the wild-type *Canton^S^* background by backcrossing *PGRP-LA^2A^* males with *Canton^S^* females for three generations in order to reduce possible effects of the genetic background.

**Figure 4:**
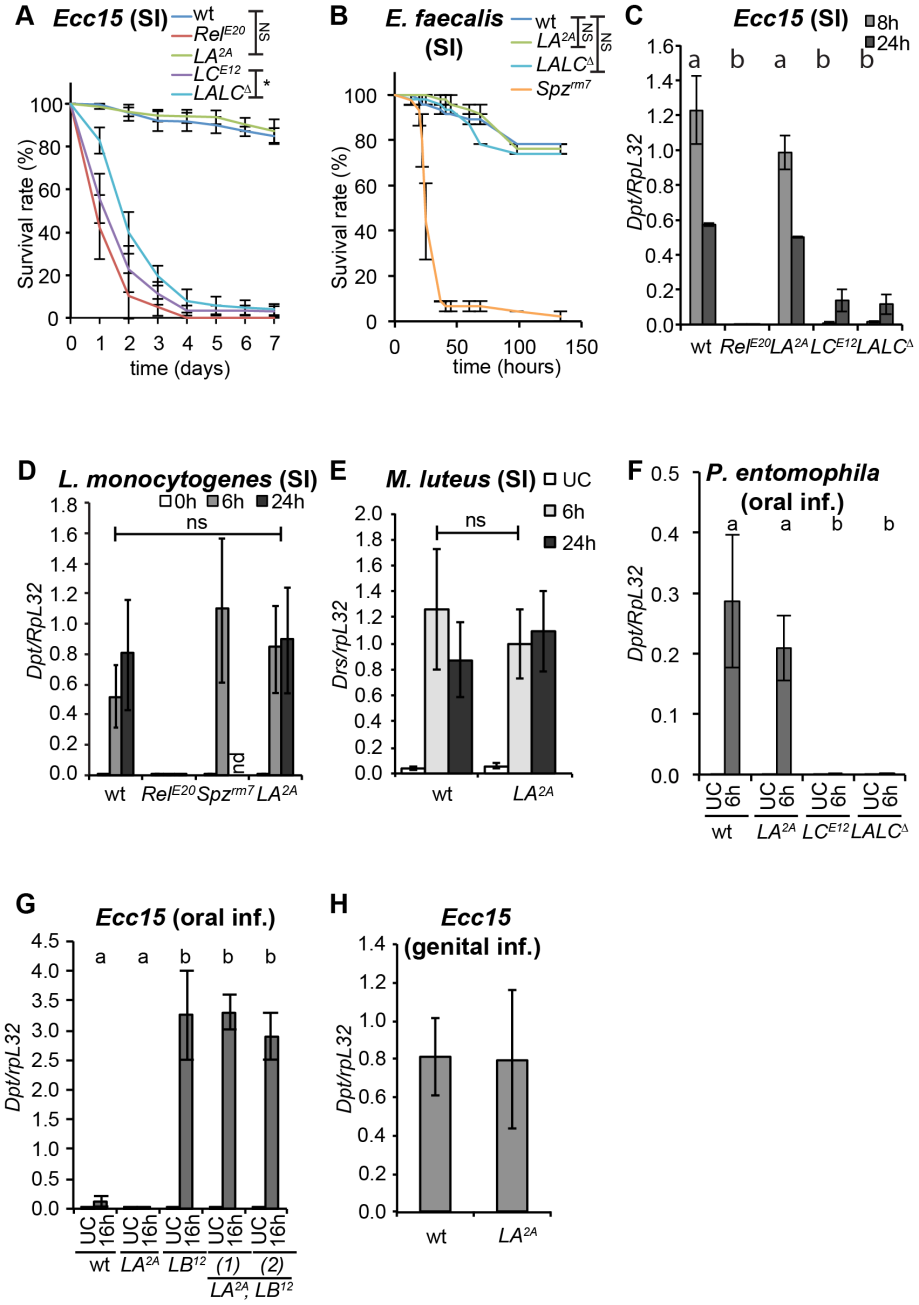
*PGRP-LA* is not required for the systemic immune response. **A,B.** Survival analysis upon septic injury with *Ecc15* in females (**A**) and *E. faecalis* in males (**B**). Full results of log-rank tests corrected with Bonferroni's method: in **A**, wt vs *Rel^E20^*: **, wt vs *LA^2A^*: ns, wt vs *LC^E12^*: **, wt vs *LALC^Δ^*: **, *LC^E12^* vs *LALC^Δ^*:*; in **B**, wt vs *Spz^rm7^*:**, wt vs *LA^2A^*: ns, wt vs *LALC^Δ^*: ns. **C–E.** RT-qPCR analysis of *Dpt* (**C, D**) and *Drs* (**E**) expression in whole females after septic injury with *Ecc15* (**C**), *L. monocytogenes* (**D**), and *M. luteus* (**E**). **F–H.** RT-qPCR analysis of *Dpt* expression in whole females after oral infection with *P. entomophila* (**F**) or *Ecc15* (**G**), and in males 6 h after genital infection by *Ecc15* (**H**). In **G**, **H**, data are shown as a ratio of LB^Δ^ 16 h (**G**) and wt (**H**). In **C–G**, data were analyzed by 2-way ANOVA with Bonferroni's multiple comparison post-tests (in **C**, **F**, **G**, a and b groups are statistically different in infected flies: *, *** and *** respectively. In **D**, wt vs *Rel^E20^* (24 h): **, wt vs *LA^2A^*: ns (*Spz^rm7^* is not included in the tests). In **F** and **G**, no significant differences were observed in unchallenged flies). In **B, C, G, H,** data are the mean of two repeats and error bars indicate data variation. In **A**, **D**, **E**, data are the mean of three independent repeats and error bars indicate standard errors. In **F**, data are the mean of 8 repeats from two independent experiments and error bars indicate standard errors. wt – wild type; *LA^2A^* – *PGRP*-*LA^2A^*; *LC^E12^* – *PGRP*-*LC^E12^*; *LALC^Δ^* – *PGRP-[LA, LC]^Δ^*; *Rel^E20^* – *Relish^E20^*; *Spz^rm7^* – *Spätzle^rm7^*. *LB^Δ^* – *PGRP-LB^Δ^*; *LA^2A^*, *LB^Δ^* (1) and (2) are two strains derived from independent recombination events between *LA^2A^* and *LB^Δ^*; nd – no data; ns: non significantly different; *,**,*** show statistical differences with p<0.05, p<0.01 and p<0.001 respectively.

As *PGRP-LA* expression is induced upon infection and as its over-expression up-regulates antibacterial gene transcription, we hypothesized that this gene was involved in the immune response. In order to clarify its role, we analyzed the effect of *PGRP-LA* deletion on the systemic immune response to different classes of microorganisms injected into the body cavity. Inactivation of *PGRP-LA* did not impact fly survival to injection with Gram-negative bacteria (*Ecc15, Salmonella typhimurium*), Gram-positive bacteria (*L. monocytogenes, Enterococcus faecalis*), or fungi (*Aspergillus glaucus*), whereas inhibition of the Imd pathway in a *Relish* mutant or the Toll pathway in a *Spätzle* mutant had a dramatic effect upon survival (**Figure 4A, B** and **Figure S2 A–C**). Consistent with these survival analyses, we did not detect an effect of *PGRP-LA^2A^* mutation on the expression levels of *Diptericin* after systemic infection with *Ecc15* in larvae and adults, infection with *L. monocytogenes* or injection of DAP-type peptidoglycan or TCT in adults, nor on levels of *Drosomycin* (a read-out of the Toll pathway) after systemic infection with the Gram-positive bacterium *Micrococcus luteus* in adults (**Figure 4C–E** and **Figure S2 D, E**). These data indicate that PGRP-LA does not function as an essential recognition receptor in either the Toll or the Imd pathway during the systemic immune response of adults. Given that *PGRP-LA* expression is enriched in epithelia, we hypothesized that this PGRP might be involved in peptidoglycan translocation and long-range activation of the systemic response observed upon oral bacterial infection in *PGRP-LB* deficient flies or upon genital infection [43], [44]. However, we did not find any role of PGRP-LA in the activation of the systemic response upon gut infections with *Ecc15* or *P. entomophila*, or genital infections with *Ecc15* (**Figure 4F–H**).

Finally, we generated a *PGRP-LA*, *PGRP-LC* double mutant in order to test if any involvement of PGRP-LA in the systemic response was masked due to a redundancy between PGRP-LA and PGRP-LC, as reported for PGRP-LE [25]. This mutant, referred to as *PGRP-[LA,LC]^Δ^*, was produced by *flp-frt* excision of a 15 kb region encompassing both genes, as depicted in **Figure 1A**. No difference in the susceptibility to infection or in the immune response activation was observed between *PGRP-[LA,LC]^Δ^* and *PGRP-LC^E12^*, a deletion containing only the *PGRP-LC* gene [22] (**Figures 4A, C, F** **and S2A, C**). We conclude that PGRP-LA does not play a major role in the systemic immune response.

### A microarray analysis reveals a role of PGRP-LA in antimicrobial genes expression in tracheae

In the absence of any overt immune function for PGRP-LA in the fat body, we next explored its role in the tracheae of larvae, a tissue in which *PGRP-LA* expression is enriched (**Figure 2A**) and up-regulated in response to infection [41]. Since the tracheal immune response is poorly characterized, we first used an unbiased approach and performed a genome-wide microarray analysis to compare the list of genes induced in the tracheae upon infection and monitor the effect of the *PGRP-LA^2A^* mutation.

**Figure 5:**
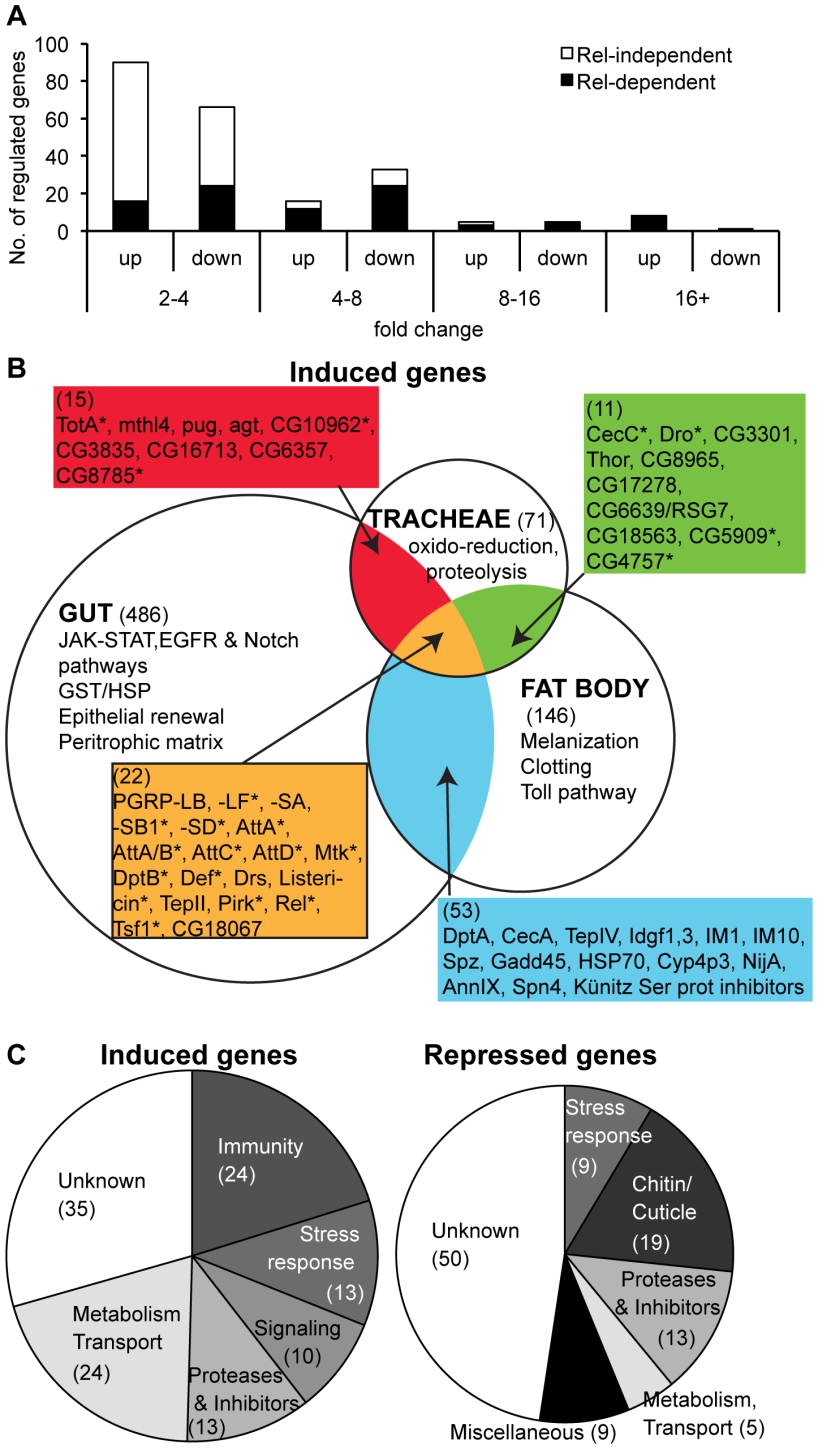
Microarray characterization of the tracheal immune response in wild-type larvae. **A.** Distribution of regulated genes, based on their up or down-regulation and their fold change in the microarray. Black and white bar portions represent the genes whose expression is affected or not affected in *Rel^E20^* respectively. **B.** Comparison of the distribution of genes up-regulated in the tracheae upon *Ecc15* bacterial infection to that of genes induced in the gut upon *Ecc15* ingestion and in whole flies upon septic injury with *Ecc15* [40], [47]. * indicates that the gene expression is affected in *Rel^E20^*. The number of genes induced in each tissue is indicated in brackets. **C.** Repartition of induced (left) and repressed (right) genes in defined categories of gene ontology.

To determine the genes specifically induced in tracheae, we investigated transcriptome variations in dissected tracheae of larvae infected with the Gram-negative bacterium *Ecc15*. We chose *Ecc15* as this bacterial strain strongly induces the Imd pathway in the tracheae upon bacterial infection, as revealed by the induction of the *Drosomycin* gene (which can be used as a read-out of Imd pathway in the trachea, see [41], [45]). The transcriptomes of wild-type, *Relish^E20^* and *PGRP-LA^2A^* third-instar larvae were analyzed in unchallenged conditions and 24h after placing larvae in *Ecc15*-contaminated fly medium at 18°C (see Materials and Methods and [46]), using Affymetrix GeneChip *Drosophila* Genome 2.0 Array. Our analysis identified 898 genes whose expression significantly varied in response to *Ecc15* infection in the wild-type strain. We focused our attention on the genes that differ by at least a 2-fold change over unchallenged condition, corresponding to 119 induced and 105 repressed transcripts, 30% of which vary by more than 4-fold (**Figure 5A**; see **Figure 5B** for a selection of up-regulated genes and **Table S1** for complete data set of regulated genes). Using a global classification, more than half of the tracheae-regulated genes were assigned to six functional categories: immunity, stress response, signaling, proteases and inhibitors, metabolism and transport, and chitin/cuticle metabolism (**Figure 5C**). Moreover, our analysis revealed a large set of previously unidentified bacteria-responsive genes, which are specific to the tracheae (71/119 and 96/105 in up and down-regulated genes respectively, **Figure 5B** and **Table S1**). To determine the contribution of the Imd pathway to antimicrobial defense in the tracheae, we examined the effect of the *Relish* mutation on gene expression. The expression of 54 up-regulated genes and 20 down-regulated genes was altered at least 2-fold in a *Relish* background compared to wild-type, with clear enrichment of Relish target genes among the most strongly induced genes (**Figure 5A** and **Table S1**). We found that 79% (19/24) of the genes annotated as immune genes were affected in the tracheae of *Relish* mutant flies (**Table S1**). Of these immune genes 71% (17/24) have been previously reported to be induced in the systemic or gut immune responses in adults [40], [47]. These genes may represent the “core” of Imd pathway-regulated genes and include *PGRP-SD, SB1 and LF*, most antibacterial peptide genes, genes coding for Imd pathway components (*Pirk*, *Relish*, *PGRP-LB*), as well as *TepII* and *Transferrin 1*.

The tracheal response to bacteria appears quantitatively less complex than the response occurring in the gut: 224 genes were modulated in the trachea using a two-fold criteria compared to 900 genes in the gut [40]. Although we cannot completely rule out an effect of the differences in stages or experimental protocols, we tend to attribute this difference to the fact that the gut response to bacteria also comprises an epithelium renewal response through stem cell proliferation and differentiation (**Figure 5B**) [40]. In the tracheae, infection induced a new set of genes notably involved in the stress response and oxidoreduction. Prominent among the repressed genes is a large set of chitin binding proteins, especially the *Twdl* family, of which 7 members are down-regulated in the tracheae, suggesting a remodeling of the highly structured intima, thin chitinous cuticle covering the tracheae [48], in response to infection. Thus, infection with *Ecc15* alters the physiology of larval tracheae, with a repression of chitin metabolism and the stimulation of immune and stress responses, as well as changes in signaling and metabolism.

**Figure 6:**
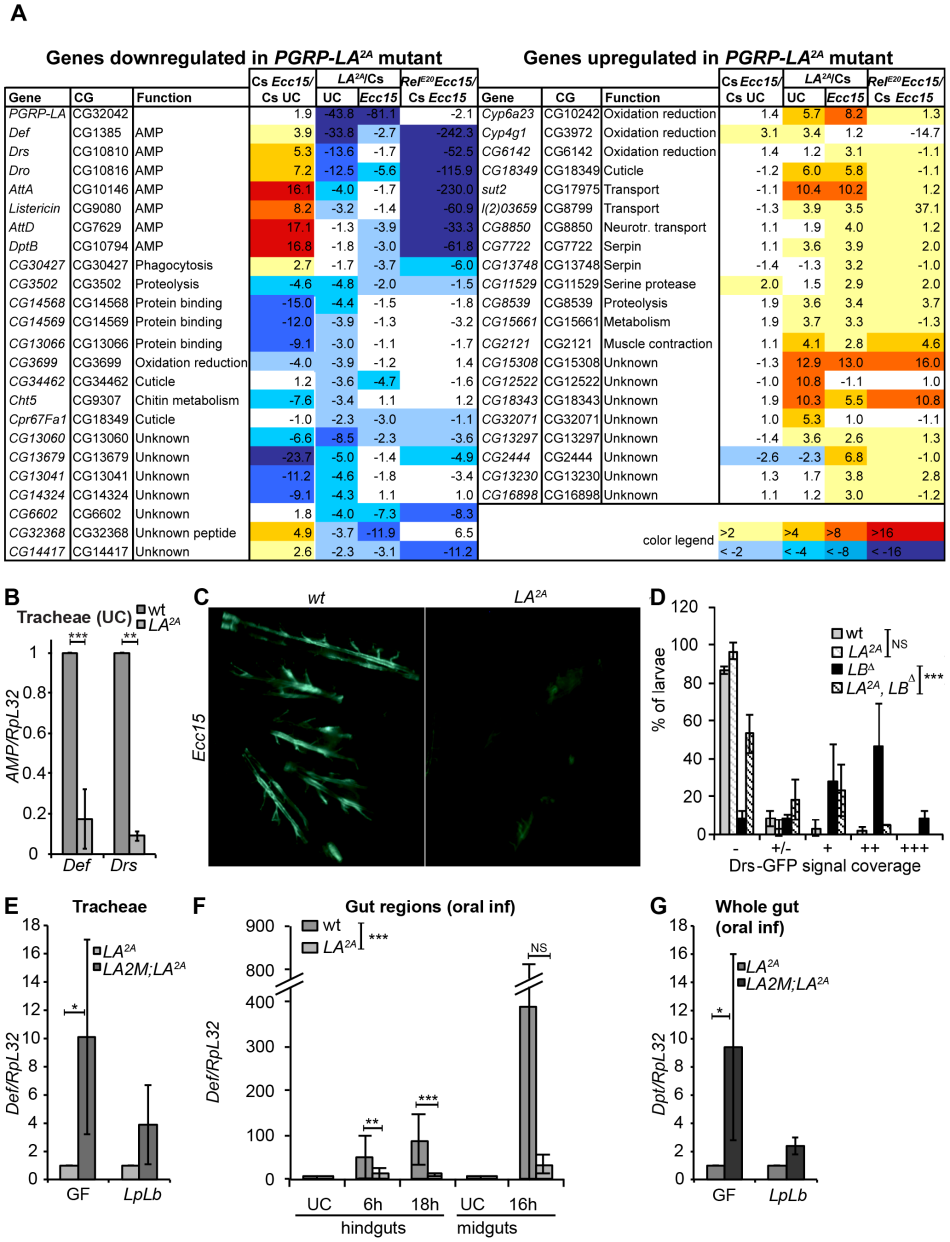
PGRP-LA promotes epithelial antibacterial responses. **A.** List of the genes that are down-regulated (left) or up-regulated (right) in the *PGRP-LA^2A^* mutant, with a fold change versus wild-type Canton S (Cs) >3 in either unchallenged or infected conditions, in the microarray analysis. For each gene the fold change in *Ecc15*-challenged versus unchallenged Cs larvae, the fold-change in *PGRP-LA^2A^* mutant versus Cs in unchallenged condition (UC) and after *Ecc15* infection, and the fold change in *Rel^E20^* versus Cs *Ecc15*-challenged larvae are provided. **B.** RT-qPCR analysis *Def* and *Drs* expression in tracheae of unchallenged wild-type and *LA^2A^* larvae. **C.** Observation of *Drs*-GFP larvae 4 days after bacterial infection with *Ecc15* at 18°C. All the larvae observed here (including of *PGRP-LA^2A^*) were showing *Drs*-GFP signal in the tracheae in more than half of the larvae, classified as ++++ in Figure S2. **D.** *Drs*-GFP signal coverage observed in unchallenged tracheae of wild-type, *PGRP-LA^2A^*, *PGRP-LB^Δ^* or *PGRP-LA^2A^*, *LB^Δ^* larvae. (−, +, ++, +++ classification is the same as in B, +/− indicates a high background level of fluorescence compared to −). Data were analyzed by grouping − and +/− on one side, +, ++ and +++ on the other side for statistical analysis. *** show statistical difference between the proportion of larvae with *Drs*-GFP signal in PGRP-*LA^2A^* vs *PGRP-LA^2A^*, *LB^Δ^* strains. **E–G.** RT-qPCR quantification after *Ecc15* infection of *Def* (**E**, **F**) and *Dpt* (**G**) expression in the larval tracheae (**E**) and in the female gut (**F**, **G**). In **E** and **G**, *LA^2A^* and *[PGRP-LA]2M;LA^2A^* lines were raised in germ-free conditions (GF) or gnotobiotic conditions (*LpLb*) where the flora is composed of *L. plantarum* and *L. brevis*. Samples were dissected 20 h after *Ecc15* infection. In **F**, wild-type and *LA^2A^* flies were conventionally reared. In **G**, it should be noted that the level of *Diptericin* was 70% higher in infected midguts of conventionally-reared [*PGRP-LA*]*2M; PGRP-LA^2A^* infected flies compared to wild-type, indicating differences in genetic background and/or microbiota between the two wild-type strains. RT-qPCR data are calibrated to unchallenged wild-type in **B**, **F** and LA2A in **E**, **G** and statistical analysis is performed prior to calibration. In **B**, data show the mean of 17 (*Def*) and 5 (*Drs*) independent experiments and error bars indicate standard errors. **C** shows data of one experiment representative of 3 independent experiments. In **D–G**, data are the mean of at least three independent experiments and error bars indicate standard error. Data analysis was performed by Mann-Whitney tests (**B**, **E**, **G**) and two-way ANOVA with Bonferroni's post-tests (**D**, **F**).

We then investigated the impact of *PGRP-LA^2A^* deletion on the transcriptome of tracheae. We confirmed that the expression of *PGRP-LA* was lost in the mutant (**Figure 6A**) and that the expression of *PGRP-LC*, which is located just upstream of the 3′ end of *PGRP-LA*, was not impaired (fold change *LA^2A^*/Cs: 1.4 both in unchallenged and infected conditions). We observed that 143 genes were more than 2-fold up- or down-regulated in *PGRP-LA^2A^* as compared to wild-type (45 of them, whose expression varies more than 3-fold threshold in the mutant, are shown in **Figure 6A**). The most significant difference between wild-type and *PGRP-LA^2A^* was the lower expression of many targets of the Imd pathway, notably antibacterial peptide genes, in both unchallenged and challenged conditions. For instance, expression of *Defensin*, *Drosomycin* and *Drosocin* were respectively 34, 14 and 13-fold lower in unchallenged *PGRP-LA^2A^* compared to wild-type larvae (**Figure 6A**). Antimicrobial peptide genes were induced in *PGRP-LA^2A^* tracheae in response to *Ecc15*, but reached a lower level than in wild-type tracheae. RT-qPCR using independent unchallenged tracheal samples confirmed that *Defensin* and *Drosomycin* transcripts were significantly lower in *PGRP-LA^2A^* compared to wild-type (**Figure 6B**).

### PGRP-LA participates in the activation of the Imd pathway in several barrier epithelia

The result above suggests a role of PGRP-LA in antimicrobial peptide gene expression in the tracheae, but not in the fat body. Nevertheless, the antimicrobial genes remain largely inducible in *PGRP-LA^2A^* mutant tracheae indicating that PGRP-LA is not a core member of the Imd pathway, but rather might participate in the fine-tuning of the epithelial immune response. It could not be fully excluded that our microarray results were caused by the genetic background (1/8^th^ of the genetic background was still different in the wild-type and mutant strains) or by an indirect influence of the microbiota that is known to influence epithelial responses [49] and could differ between the two strains. Thus, we repeated and extended experiments using additional strains and conditions. We first monitored the level of *Drosomycin*-GFP in wild-type and *PGRP-LA^2A^* tracheae of unchallenged and *Ecc15*-infected larvae. After infection with *Ecc15*, we observed that the proportion of *Drosomycin-GFP* expressing larvae was smaller in *PGRP-LA^2A^* mutants (**Figure S3**) and that even when selecting larvae expressing the reporter, the fluorescence intensity was lower in *PGRP-LA^2A^* mutant than in wild-type tracheae (**Figures S3,** **6C**). The difference was less clear in unchallenged conditions, as the expression of *Drosomycin-GFP* was very low, even in the wild-type (**Figure 6D**). Thus, we decided to use a fly line deficient for *PGRP-LB*, which encodes a negative regulator of the Imd pathway [18]. As reported before, tracheae of larvae where *PGRP-LB* is down-regulated express a much higher level of *Drosomycin-GFP* reporter compared to wild-type [17]. We observed that the GFP signal in double mutant *PGRP-LA^2A^*, *LB^Δ^* larvae was significantly more restricted than in *PGRP-LB^Δ^* larvae (**Figure 6D**).

To confirm that the effect seen on the activation of the Imd pathway was not due to the genetic background, we also performed a genomic rescue of the *PGRP-LA* deficiency line with a transgene containing the *PGRP-LA* locus including 4 kb upstream of the start codon (referred to as *[PGRP-LA]2M*). In both the tracheae and the midgut, the expression of *PGRP-LA* in the rescue line (genotype: [*PGRP-LA*]*2M; PGRP-LA^2A^*) was similar to wild-type levels (**Figure S1B, C**). In order to elude any effect of the microbiota, we generated axenic (germ-free) *PGRP-LA^2A^* and [*PGRP-LA*]*2M; PGRP-LA^2A^* lines, reconstituted a gnotobiotic microbiota composed of *Lactobacillus plantarum* and *Lactobacillus brevis* only, two bacteria commonly found in *Drosophila* microbiota (reviewed in [50]) and maintained these germ-free and gnotobiotic lines in autoclaved fly medium. In these conditions, the levels of *Drosomycin* and *Defensin* in unchallenged tracheae were very low and too variable, preventing us to analyze the effect of the *PGRP-LA^2A^* deletion on basal Imd pathway activation by RT-qPCR. We therefore focused our analysis on tracheae of larvae collected 24 h after bacterial infection with *Ecc15* at 29°C. *Defensin* and *Drosomycin* expression was 3 to 10-fold lower in the tracheae of *PGRP-LA^2A^* compared to *[PGRP-LA]2M; PGRP-LA^2A^* infected larvae (**Figures 6E** **, S4**). The effect of *PGRP-LA* on tracheal antimicrobial genes upon *Ecc15* infection was still observed when larvae were raised in germ-free conditions. The results were however variable and statistical significance could only be observed when monitoring *Defensin* after infecting germ-free larvae (**Figures 6E**).

To extend our analysis, we next investigated whether PGRP-LA was involved in the Imd pathway activation in the gut of adults since *PGRP-LA* is also enriched in this tissue (**Figure 2A**). **Figure 6F** shows that the level of *Defensin* was significantly lower in the hindgut of *PGRP-LA^2A^* flies as compared to wild-type following oral infection with *Ecc15*. The effect of PGRP-LA on *Defensin* expression was less marked in the midgut. To confirm this result, we also monitored *Diptericin* expression in the gut of *PGRP-LA^2A^* and *[PGRP-LA]2M; PGRP-LA^2A^* adult flies raised in either germ-free or gnotobiotic conditions, and then infected with *Ecc15*. **Figure 6G** shows that *Diptericin* expression was also lower in the gut of *PGRP-LA^2A^* mutant compared to [*PGRP-LA*]*2M; PGRP-LA^2A^* adults 20 h after oral infection with *Ecc15* although this effect was only significant when infecting previously germ-free flies (**Figure 6G**).

Together with the microarray analysis, these data suggest that PGRP-LA positively regulates the Imd pathway in barrier epithelia such as the tracheae and the gut.

## Discussion

In this manuscript, we present a first detailed analysis of PGRP-LA function. Our structural study predicts that the PGRP domain of PGRP-LA is unlikely to bind peptidoglycan by itself. We next show that over-expression of *PGRP-LA_D_* isoform, but not of *PGRP-LA_C_* and *PGRP-LA_F_*, leads to the activation of *Diptericin* expression in absence of infection. Our experiments placed PGRP-LA_D_ upstream of the Dredd caspase and of the Tak1 MAP3K. The intracellular domain of PGRP-LA_D_ contains a RHIM motif similar to that observed in PGRP-LC and PGRP-LE for which it is essential for Imd pathway activation [26]. This suggests that the RHIM motif confers to PGRP-LA_D_ the capacity to induce the Imd pathway. Studies involving short mutations in PGRP-LC and PGRP-LE reported that their RHIM motifs are not involved in any physical interaction with Imd, the downstream adaptor of the Imd pathway, but bind with Pirk, a negative regulator of the Imd pathway [26], [51]. Further analysis will be required to test whether the different PGRP-LA isoforms physically interacts with Pirk and/or with PGRP-LC. Collectively, this initial molecular characterization of PGRP-LA suggests a modulatory role of this PGRP in the Imd pathway.

Using a *PGRP-LA*-deficient line, we showed that *PGRP-LA* is not required for the systemic production of antimicrobial peptides in the adult. Consistent with this observation, mutations in *PGRP-LA* did not increase the susceptibility to systemic bacterial infection. This matches with the very low expression of *PGRP-LA* in the fat body. Of note, phagocytosis was also not affected in the *PGRP-LA^2A^* mutant (**Figure S5**). Consistently, previous studies on S2-cells did not reveal any role of PGRP-LA in the induction of antimicrobial peptides by peptidoglycan or Gram-negative bacteria [23], [24] or in the phagocytosis of Gram-negative or Gram-positive bacteria [24]. All these data clearly indicate that PGRP-LA is not compulsory for the systemic activation of the Imd or Toll pathways, although a more specific role under a very specific condition or in response to a specific form of peptidoglycan could formally not be excluded.

Several studies have shown that the antimicrobial response of *Drosophila* exhibits major differences depending on the tissue [1], [40], [45], [52], [53]. Notably, regulatory mechanisms controlling the antimicrobial response in barrier epithelia significantly differ from that involved in fat body-mediated systemic immune response. For instance, the expression of antimicrobial peptide genes (including *Drosomycin*) in the midgut or the tracheae relies only on the Imd pathway [45]. In addition, it has recently been shown that PGRP-LE has a significant role in Imd pathway activation in the midgut while PGRP-LC is the main sensor of Gram-negative bacteria during systemic infection [27], [28]. These differences are probably a consequence of the necessity to maintain tight control on immune activation according to the level of exposure to bacteria or microbial products; while the hemocoel surrounding the fat body remains sterile, organs such as the digestive tract and tracheae are constantly in direct contact with the external environment. This raises the possibility that PGRP-LA has a subtler role in barrier epithelia where its expression is enriched. In support of this notion, our microarray analysis revealed a lower expression of antimicrobial peptides in *PGRP-LA^2A^* tracheae of both *Ecc15*-infected and unchallenged larvae. The idea that PGRP-LA could establish the basal level of Imd pathway in unchallenged conditions is intriguing. These results were confirmed in RT-qPCR (**Figure 6B**), but limitations due to the low and variable levels of antimicrobial gene expression in the tracheae and the gut in unchallenged conditions, when maintaining fly lines in autoclaved fly medium, did not allow us to confirm this hypothesis (data not shown). Nevertheless, we observed that the expression of several antimicrobial peptide genes was reduced in larval tracheae and adult guts of *PGRP-LA^2A^* mutants upon *Ecc15* infection. A rescue experiment confirms that the phenotype is specifically linked to the *PGRP-LA* deletion and not to the genetic background. However, in normal laboratory conditions the PGRP-LA phenotype is not very strong and we were unable to detect any infectious condition for which a contribution of PGRP-LA to adult survival was discernable.

Our results support the notion that PGRP-LA positively regulates the antibacterial response in infected epithelia. However, we cannot exclude subtle additional roles for PGRP-LA, such as its participation in inter-organ communication by spreading immune signaling from epithelia to another tissue (e.g. between the gut and the tracheae). Such immune communication between tissues occurs between several epithelia and the fat body in *Drosophila* [17], [44], [46], [54]. However, no role of PGRP-LA could be discerned in the activation of the systemic response upon gut or genital infections (**Figure 4F–H**).

The implication of several pattern-recognition receptors in the gut highlights the complexity of mechanisms underlying bacterial sensing in barrier epithelia. The conservation of PGRP-LA in mosquito (contrary to PGRP-LE or PGRP-LF) where it is also located in cluster with PGRP-LC suggests the conservation of its function in other insect species. The genomic organization of the PGRP-LA, LC, LF cluster is intriguing since the Imd-receptor gene *PGRP-LC* is flanked by both a positive (*PGRP-LA*) and a negative (*PGRP-LF*) regulator of the pathway. Future studies should elucidate the mechanisms by which PGRP-LA modulates the Imd pathway, notably to determine which PGRP-LA isoforms are involved. Another question to address will be the respective contributions of PGRP-LA, LC, and LE in the sensing of bacteria in the intestine. Thus, our data add a layer of complexity to the mechanism regulating the Imd pathway and further investigation is needed to fully characterize the role of PGRP-LA.

The Drosophila tracheal immune response remained poorly characterized [41], [55], [56]. In this study, we also present a general analysis of tracheal transcriptome variations after bacterial infection in larvae. Our data reveal a major role of the Imd pathway, which controls the expression of half of the genes regulated upon infection and of most of the immunity-related genes, such as antimicrobial genes. This is in accordance with previous reports showing that this pathway controls the local production of antimicrobial peptide genes, in tracheae and the gut [40], [45], [57]. We note that it also regulates genes involved in other cellular functions such as metabolism. Interestingly, we observed that many genes encoding putative or characterized cuticle proteins are down-regulated upon infection. The shape of the tracheae is maintained by helicoidal thickenings of the intima called taenidiae [48]. Therefore, the down-regulation of structural genes highlighted in our microarray suggests a remodeling of this structure upon infection. Consistent with this down-regulation, an apical-basal enlargement of the cells of the airway epithelium has been previously reported in regions of the tracheae exhibiting a strong immune response [41]. This enlargement might be explained by a thinning of the cuticle and consequent loss of rigidity. Thus, infection with *Ecc15* not only induces an immune and stress response, but also alters the metabolism and physiology of tracheae. Interestingly, microarray comparison of the immune response during systemic (fat body), gut, and tracheal immune response reveals that only a small group of common genes are induced, all regulated by the Imd pathway and encoding mainly antimicrobial peptides and other pathway components. These genes may therefore represent the “core” of Imd pathway that are complemented by tissue-specific genes to achieve an optimal immune response.

## Materials and Methods

### Fly Stocks

*Oregon^R^* flies were used as wild-type controls for the *PGRP-LA^2A^* original strain (**Figure 4A, H** **,** **6H, S** **2B**), and *Canton^S^* flies were used as wild-type controls of *PGRP-LA^2A^* introgressed into the *Canton^S^* background (all other figures). Over-expression experiments were controlled by crossing the *da-Gal4* driver to *w^1118^*, the strain in which the UAS construct insertions were generated. *Relish^E20^ (e^+^, Rel^E20^)*, *Dredd^B118^*, *Tak1^1^*, *PGRP-LC^E12^* and *Spätzle^rm7^* are described elsewhere [22], [58]–[61]. The *da-Gal4* line expresses Gal4 ubiquitously and constitutively. The *UAS-PGRP-LA_C_* (insertion R1), *UAS-PGRP-LA_D_* (insertion R4) and *UAS-PGRP-LA_F_* (insertion R2) lines were obtained as follows. A full-length cDNA of each isoform of *PGRP-LA* (using the *CG32042*_cDNA gold GH4960, GH18280 and GH10945, respectively, from DGRC) was placed downstream of the UAS sequence using the pUASt vector. F1 progeny young adults carrying both the *UAS* construct and the *Gal4* driver were transferred to 29°C for optimal efficiency of the UAS/Gal4 system.

Stocks were reared at 25°C on media prepared as follows: per liter of water, 58.8 g inactivated yeast (Biospringer Springaline® BA95/0), 58.8 grams maize flour (Westhove Farigel Maize H1), 7.5 g agar, 58 mL of 1∶1 mix of grape and multi-fruit juice were combined with water and boiled at 80°C for 30 min. When the mixture had cooled to 65°C, 4.85 ml of 99% proprionic acid and 30 ml of a 10% solution of methyl paraban in 85% ethanol were added. After cooling to room temperature, live yeast was added on the surface of the media, except for germ-free and gnotobiotic flies, which were reared on autoclaved media in glass tubes without the addition of live yeast.

*PGRP-LA^2A^* mutant was obtained by imprecise excision of the G14937 P-element (KAIST library) and *PGRP-[LA,LC]^Δ^* by FRT mediated deletion of the region between the P-elements 1930 and 4396, following previously published methods [62]. *[PGRP-LA]2M* rescue line was generated using gap-repair and recombineering, and final rescue construct carried by P[acman] vectors was inserted into the PhiC31 landing site 51C on chromosome 2 (BDSC strain 24482) [63],[64]. Vector with *PGRP-LA* contain the *PGRP-LA* gene including the following sequence (based on Flybase release r5.47): 3L: 9323736–9331619.

*Drosophila* stocks and crosses were maintained at 25°C in yeasted tubes containing corn-meal fly medium. Germ-free lines were generated by egg bleaching and kept in autoclaved fly medium. Gnotobiotic lines were generated by introducing cultured *L. plantarum* and *L. brevis* previously isolated from our fly lines into the medium of germ-free lines and were also kept in autoclaved fly medium.

### Bacterial and fungal stocks

All bacteria were stored as frozen stocks (15% DMSO). *Erwinia carotovora carotovora 15* (*Ecc15*), *Escherichia coli*, *Enterococcus faecalis*, *Pseudomonas entomophila*, and *Micrococcus luteus* were described previously [61]. They were cultured on LB-Agar plates and grown overnight in LB-medium at 29°C and generally used as pellets of OD_600_ = 200, i.e. the OD_600_ of a 1/1000^th^ dilution of the pellet in PBS was 0.2 corresponding to 4.10^11^ CFU.mL^−1^ (exceptions are mentioned below). *Salmonella typhimurium*, *Listeria monocytogenes* and *Candida albicans* were described previously [61]. They were grown overnight at 37°C, respectively in LB-medium, BHI, and YPG. *Aspergillus glaucus* was kept as a spore-suspension at 4°C and injected as such into the flies. While testing the susceptibility to septic injury *Ecc15* was tested at OD_600_ 200 and 50, *Salmonella typhimurium* at OD_600_ 0.65 and 10^−5^, *L. monocytogenes* at OD_600_ 0.65 and 10^−5^, *Enterococcus faecalis* at OD_600_ 5, 10, 15.

### Infection and survival experiments

Septic injuries were made by pricking adults in the thorax with a thin needle dipped into a concentrated bacterial pellet. Genital infections were performed by touching the tip of the abdomen with a 200 µL pipette-tip containing 10 µL of bacterial pellet [44]. For gut infection, flies were starved for 2 h, then allowed to feed on a 1∶1 mixture of 5% sucrose and concentrated bacteria (OD_600_ = 200), peptidoglycan (5 mg/ml), or TCT (tracheal cytotoxin; 0.046 mM) applied to a filter disk completely covering the surface of standard fly medium. Flies were maintained at 29°C and guts were dissected 16–24 h after contact with infected food. In **Figures 6F** **, S1**, midgut was defined as the section of the gut between the proventriculus (included) and the pylorus (midgut/hindgut junction), while hindgut corresponds to the section between the pylorus and the anus. Malpighian tubules were excluded from both midgut and hindgut samples. In **Figure 6G**, whole guts include the section between the crop (included) and the anus, malpighian tubules being removed. A minimum of 20 flies were used for survival experiments. Survival was scored once to twice a day [**61**] and data were analyzed by logrank tests corrected with Bonferroni's method.

To monitor the immune response in the tracheae, two different methods of infection were used. *Method 1* (Microarray, **Figures 5** **,** **6A, C, D** **, S3**): adults were allowed to lay eggs for 3 days then removed, and 500 µL *Ecc15* pellet (OD_600_ = 200) were added on the 4^th^ day into 28.5 mm-wide vials where larvae were developing. The vials were then put at 18°C for 24 h and non-wandering 3^rd^-instar larvae (with hand-shaped anterior spiracles) were dissected. *Method 2* (**Figure 6E** **and S4**): larvae were incubated for 30 min in a 2-mL tube with a 1∶1 mixture of mashed banana and *Ecc15* (OD_600_ = 200), then the tube content was transferred in a fresh fly vial and kept at 29°C [**61**].

### RT-qPCR

Antimicrobial peptide genes and *RpL32* mRNA quantification by RT-qPCR was performed as described previously [61]. For *PGRP-LA*, the primers were designed to amplify a region included in all isoforms and in the deleted part of *PGRP-LA^2A^* to allow both expression quantification and deletion control (sequences of the qPCR primers: Fp: CCT-TTA-TGG-GCG-ACT-ATG-GC and Rg: CTT-GGC-GTC-CCA-CGA-TTC) (**Figure 1A**). Unless otherwise noted, all expression data are given as a ratio of the expression level of the invariant mRNA *RpL32*. Each experiment was performed with approximately 20 flies for each genotype.

### Microarray Analysis

Larvae were infected with method 1 and dissected by gently pulling the posterior spiracles backwards until the whole tracheae went out. If needed, the anterior part of the tracheae was pulled out in a second similar step. RNA pools from the tracheae (including anterior spiracles) of 50 3^rd^ instar larvae were isolated, purified with RNA clean-up purification kits (Macherey Nagel), and DNase treated. The samples were controlled for fat body contamination by RT-qPCR on *Fat body protein P6* (*Fbp2*). RNA quality was controlled on Agilent 2100 Bioanalyzer chips. As the quality of some samples was not good enough after this first purification, RNA of all samples was ethanol precipitated to pass Bioanalyzer quality control. For each sample, 100 ng of total RNA was amplified and labeled using the GeneChip IVT Labeling Kit according to the protocol provided by the supplier. Affymetrix Drosophila Genome 2.0 arrays were hybridized with 30 mg of labeled cRNA, washed, stained, and scanned according to the protocol described in the Affymetrix Manual. Three independent repeats were performed for each condition and gene expression profiles from challenged larvae were normalized to their controls. Statistical analyses were performed using the R and Bioconductor statistical packages. Full dataset can be found at http://lemaitrelab.epfl.ch/ and has been deposited at EMBL-EBI database (Accession number: E-MEXP-3925).

### Imaging

For GFP observation, flies were dissected in PBS and either directly observed under a Leica MZ16F dissecting microscope, or mounted in PBS for imaging with a Zeiss Axioimager Z1. Images were captured with a Leica DFC300FX camera and Leica Application Suite or with an Axiocam MRn camera and Axiovision respectively.

### Phagocytosis assay

The phagocytosis assay was performed as previously published [65]. Briefly, 41.1 nL of *S. aureus* or *E. coli* bioparticles (20 mg.mL^−1^) were injected in the fly abdomen. Flies were left for 30–40 min at 25°C and injected with 6×69 nL of 0.4% trypan blue.

### Accession Numbers

The Flybase (www.flybase.org) accession numbers for genes mentioned in the microarray are indicated in the data (**Figure 6A** **, Table S1**). The accession numbers for genes mentioned in the rest of this study are: *Defensin* (CG1385), *Diptericin* (CG12763), *Drosocin* (CG10816), *Drosomycin* (CG10816), *PGRP-LA* (CG32042), *PGRP-LB* (CG14704), *PGRP-LC* (CG4432), *PGRP-LF* (CG4437), *da* (*daughterless*, CG5102), *pirk* (CG15678) and *Relish* (CG11992). The vectorbase (www.vectorbase.org) accession numbers for Culex and Anopheles homologs of PGRP-LA are CPIJ006558 and AGAP005205 respectively.

## Supporting Information

Table S1 **Expression profile of the genes regulated in the tracheae upon bacterial infection in larvae.** List of the genes showing a fold change >2, upon *Ecc15* infection, in the tracheae of *Canton^S^* larvae. Fold changes in *Canton^S^* and *Rel^E20^* are indicated. In the “Rel” column, “R” indicates the genes whose regulation is affected in *Rel^E20^* mutant. The columns “sys”, “gut” and “sys+gut” show respectively the genes regulated in whole flies upon septic injury with *Ecc15* [47], in the gut upon *Ecc15* ingestion [40], and in both conditions; for each tissue,“+” means that the gene is up-regulated “−” that it is repressed. AvgExp: mean signal over all chips.
(PDF)

Figure S1 ***PGRP-LA*** **expression in tissues.** RT-qPCR analysis of *PGRP-LA* expression in wild-type adult female tissues (**A**) and in adult female midguts (**B**) and larval tracheae (**C**) of wild-type and *[PGRP-LA]2M; PGRP-LA^2A^* strains. Data are normalized to *RpL32* and shown as a ratio of the expression in the wild-type. In **A**, a single experiment was performed. In **B**, data are the mean of three independent experiments, error bars indicate standard errors and data were analyzed by 2-way ANOVA with Bonferroni post-tests. In **C**, data are the mean of two independent experiments and error bars indicate data variation.
(TIF)

Figure S2 ***PGRP-LA*** **is not required for the systemic immune response.** **A–C.** Survival analysis of flies after injection with *S. typhimurium* (**A**, OD 10^−5^, 69 nL injected), *L. monocytogenes* (**B**, OD 6.5, 9.2 nL injected), *A. glaucus* (**C**, spore suspension, 69 nL injected). **D,E.** *Dpt* expression after injection of 9.2 nL of monomeric (tracheal cytotoxin, TCT, 0.46 mM) or polymeric peptidoglycan (PGN, 5 mg.mL^−1^) (**A**) and after septic injury with *Ecc15* in larvae (**B**).
(TIF)

Figure S3 **Tracheal** ***Drs*** **response in wt and** ***PGRP-LA^2A^*** **deficient larvae (GFP).** Fluorescence observed in the tracheae of wild-type and *PGRP-LA^2A^* larvae expressing the *Drs*-GFP reporter gene 4 days after bacterial infection with *Ecc15* at 18°C. (−) no fluorescence (+) fluorescence in the spiracles only, (++) in the tracheal trunks, (+++) in the tracheae in less than half of the larva or (++++) in the tracheae in more than half of the larva. •, ••, •••: increasing intensity of fluorescence. Data of one experiment representative of 3 independent experiments are shown.
(TIF)

Figure S4 **Tracheal** ***Drs*** **response in wt and** ***PGRP-LA^2A^*** **deficient larvae (RT-qPCR).** RT-qPCR quantification of *Drs* expression in the larval tracheae 24 h after *Ecc15* infection in *LA^2A^* and *[PGRP-LA]2M;LA^2A^* lines raised in germ-free conditions or gnotobiotic conditions (*LpLb*) where the flora is composed of *L. plantarum* and *L. brevis*. Data show the mean of 4 repeats and error bars indicate standard errors. Data were analyzed by Mann-Whitney tests, differences are non significant.
(TIF)

Figure S5 ***PGRP-LA*** **is not required for the phagocytosis.** Fluorescent images of fly abdomens after injection of *S. aureus* or *E. coli* nanoparticles. Data show representative results of one experiment.
(TIF)
