# Supplementary material for: Functional Analysis of PGRP-LA in Drosophila Immunity
Source: PLoS One. 2013 Jul 26;8(7):e69742. doi: 10.1371/journal.pone.0069742 (PMC3724876; doi:10.1371/journal.pone.0069742)
Supplement: Table S1 — Expression profile of the genes regulated in the tracheae upon bacterial infection in larvae. List of the genes showing a fold change >2, upon Ecc15 infection, in the tracheae of CantonS larvae. Fold changes in CantonS and RelE20 are indicated. In the “Rel” column, “R” indicates the genes whose regulation is affected in RelE20 mutant. The columns “sys”, “gut” and “sys+gut” show respectively the genes regulated in whole flies upon septic injury with Ecc15 [47], in the gut upon Ecc15 ingestion [40], and in both conditions; for each tissue,“+” means that the gene is up-regulated “−” that it is repressed. AvgExp: mean signal over all chips. (PDF) [file pone.0069742.s001.pdf]

Gene.Title	Gene.Symbol	CG	Rel	sys.	gut	sys+ gut	CsEcc15	RelEcc15 vs CsUC	RelUC vs CsUC	RelEcc15 vs RelUC	AvgExpr
**INDUCED**	**119**										
**IMMUNITY**			Total	Total	Total	Total					
**Antimicrobial peptides genes**			92	35	42	22					
		induced	39	33	37	22					
Metchnikowin	**Mtk**	CG8175	R	+	+	+	57.2	1.1	-1	1.1	3.5
attacin	**AttC**	CG4740	R	+	+	+	23.4	-1.8	-1.7	-1.1	5.9
Cecropin	**CecC**	CG1373	R	+			18.9	-1.1	-1.1	1	2.7
attacin	**AttD**	CG7629	R	+	+	+	17.1	-2	-2	1	4.7
diptericin-like protein	**DptB**	CG10794	R	+	+	+	16.8	-3.7	-3.7	-1	6.9
attacin /// attacin	**AttA /// AttB**	CG18372	R	+	+	+	16.3	-5.8	-6	1	7.9
attacin	**AttA**	CG10146	R	+	+	+	16.1	-14.3	-11.8	-1.2	7.7
drosocin	**Dro**	CG10816	R	+			7.2	-16	-14.2	-1.1	7.8
drosomycin	**Drs**	CG10810		+	+	+	5.3	-9.8	-87.6	8.9	11.4
defensin	**Def**	CG1385	R	+	+	+	3.9	-62.4	-70.7	1.1	11.3
**Defense reaction**											
Transferrin 1	**Tsf1**	CG6186	R	+	+	+	3.6	-1.1	1.3	-1.4	9.6
virus-induced RNA 1	**vir-1**	CG31764					2.1	4	1.8	2.3	9
**Recognition**											
PGRP-SD	**PGRP-SD**	CG7496	R	+	+	+	7.3	-2.3	-1.9	-1.2	5.6
PGRP-SB1	**PGRP-SB1**	CG9681	R	+	+	+	6.7	-2	-1.4	-1.4	6.5
PRGP-like	**PGRP-LF**	CG4437	R	+	+	+	5.3	1.8	-1.5	2.6	8
Peptidoglycan recognition protein LB	**PGRP-LB**	CG14704		+	+	+	2.4	1.8	1	1.7	5.4
semmelweis	**PGRP-SA**	CG11709		+	+	+	2	1.5	1.1	1.4	10.1
Thiolester containing protein I	**TepI**	CG18096	R				4.8	2.8	5.1	-1.8	4
Thiolester containing protein II	**TepII**	CG7052		+	+	+	2.9	1.3	-1.1	1.5	8.3
Lectin28C /// rhodopsin	**lectin-28C /// ninaE**	CG7106	R	-			2.5	-1.6	-1.7	1.1	6.3
galactin	**Lectin-galC1**	CG9976	R				2.4	-5.9	-3.2	-1.8	6.6
**Imd pathway**											
CG15678	**pirk**	CG15678	R	+	+	+	3.8	1.1	1.6	-1.5	5.6
relish	**Rel**	CG11992	R	+	+	+	2.7	-37	-42.9	1.2	9.5
**Phagocytosis**											
CG30427	**CG30427**	CG30427	R				2.7	-2.2	-2.5	1.1	7.1
**STRESS RESPONSE/DETOXIFICATION**											
**Stress response**											
Turandot A	**TotA**	CG31509	R		+		21.3	28.3	8.3	3.4	4.3
Cyp312a1	**Cyp312a1**	CG5137					2.7	4.9	1.5	3.1	6.6
Heat shock protein cognate 1	**Hsc70-1**	CG8937					2.6	-1	-1.6	1.6	5.7
Cyp9f2	**Cyp9f2**	CG11466					2.3	3.5	1.7	2	8.9
Cyp4d21	**Cyp4d21**	CG6730					2.4	-9.9	-13.6	1.4	7.7
Cytochrome P450-4e1	**Cyp4e1**	CG2062					2	4.7	3.5	1.4	6.1
Cytochrome P450-9b1	**Cyp9b1**	CG4485			-		2	2.1	1.4	1.5	7.6
Cyp313a2	**Cyp313a2**	CG10094					2	4.4	2.4	1.8	7.8
Cyp4p2	**Cyp4p2**	CG1944					2.2	-2.4	-2.9	1.2	6.3
**ROS detoxification**											
CG10962	**CG10962**	CG10962	R		+		7.3	1	-1.3	1.4	6.3
CG12466 /// CG40486	**CG12466 /// CG40486**	CG40486					3.2	7	3.2	2.2	3
CG3835	**CG3835**	CG3835			+		2.1	1.8	-1.1	2	10
CG3301	**CG3301**	CG3301		+	-		2.1	1.4	-1.3	1.8	5.9
**SIGNALING**											
**Insulin pathway**											
insulin-stimulated eIF-4E binding prote	**Thor**	CG8846		+			2.2	3.1	1.5	2.1	11.1
insulin receptor	**InR**	CG18402					2	2.3	-1	2.3	5.7
**Signaling**											
medullaless	**ana**	CG8084					2.8	3.7	1.1	3.4	4.7
methuselah-like 4	**mthl4**	CG6536			+		2.5	3.1	1.5	2	8
CG11438	**CG11438**	CG11438					2.3	4.7	1.9	2.5	6.8
CG8334	**CG8334**	CG8334					2.4	1.3	1.1	1.2	6
CG11919 /// CG18003	**CG11919 /// CG18003**	CG30019					2.3	-1.3	-2.3	1.7	6.4
CG8965	**CG8965**	CG8965		+			2.1	2.2	1.6	1.4	6.1
focal adhesion kinase	**PR2**	CG3969					2.1	1.2	1	1.2	8.5
CG17278	**CG17278**	CG17278		+			2	-1.3	-1.3	1	11.1
**PEPTIDASE&INHIBITOR**											
**Serine protease**											
CG11668	**CG11668**	CG11668	R				6	1	1.1	-1.1	5.6
CG6639 /// Regulator of G-protein sign	**CG6639 /// RSG7**	CG6639		+			5.6	2.4	-1.6	4	6.3
CG18563	**CG18563**	CG18563		+			3.3	1.8	-1.4	2.5	5.7
CG5909	**CG5909**	CG5909	R	+			2.7	-1.5	-1.7	1.1	5.4
CG17572	**CG17572**	CG17572					2.2	4.2	2.7	1.5	6.6
CG11313	**CG11313**	CG11313	R				2.1	-1.9	-1.8	-1	6.5
CG5896	**grass**	CG5896					2.1	1.2	1	1.2	10.1
CG11529	**CG11529**	CG11529					2	4	-1	4.1	7.7
**Serine-type endopeptidase inhibitor activity**											
CG16713	**CG16713**	CG16713		+	+	+	2.7	-1.6	-4.7	3	7.7
CG6663	**CG6663**	CG6663	R	-			2.3	1	1.1	-1	5.1

**Other peptidase**												
CG5863	**CG5863**	CG5863					3.5	1.5	-1.4	2.1	7.1
CG6357	**CG6357**	CG6357		+	+	+	3.4	4	1.8	2.2	6.1
CG13283	**CG13283**	CG13283					2.1	2.1	-1	2.1	5.3
**METABOLISM/TRANSPORTERS**												
**Transporters**												
CG8785	**CG8785**	CG8785	R		+		4.6	-1.6	-2.2	1.4	7.3
CG4607 /// CG4607	**CG4607 /// DsimCG4607**	CG4607	R				2.7	-1.6	-1.2	-1.4	8.7
CG8925	**CG8925**	CG8925			-		2.2	2	1.2	1.7	7.5
CG4484	**CG4484**	CG4484					2.1	3.1	2.4	1.3	7.6
Epidermal stripes and patches	**Esp**	CG7005					2.1	-1.2	-2.2	1.9	6.7
**Metabolism**												
CG4716 /// CG4716	**CG4716 /// DsimGD10957**	CG4716		-			11.7	3.4	-7.1	24.3	7
CG4757	**CG4757**	CG4757	R	+			7.9	94.9	147.3	-1.6	4.8
ornithine decarboxylase	**Odc1**	CG8721		-			3.2	2.5	-1.2	3	5.3
CG6461 /// CG6461	**CG6461 /// DmirCG6461**	CG6461	R				3	1.1	1.1	-1	7.6
CG18473	**CG18473**	CG18473	R				2.9	2.1	1.7	1.3	5.2
CG9452	**CG9452**	CG9452					2.7	1.4	-1.4	1.9	6.3
UTP-G-1-P uridyiyltransferase	**UGP**	CG4347		-			2.6	-2.1	-3.3	1.5	7.3
lysyl oxidase-like 2	**lox2**	CG4402					2.6	2.4	1	2.2	7.6
Sorbitol dehydrogenase-2	**Sodh-2**	CG4649					2.5	4.2	1.8	2.3	9.7
CG18067	**CG18067**	CG18067		+	+	+	2.4	3.4	2.3	1.5	10.2
CG30359	**CG30359**	CG30359					2.4	1.6	-1.1	1.9	8.9
Acetyl CoA synthetase	**AcCoAS**	CG9390					2.4	1.1	-1.4	1.5	9.8
Pugilist	**pug**	CG4067			+		2.2	1.2	-1.6	1.8	7.6
CG5431	**CG5431**	CG5431					2.1	4.1	3.8	1.1	7
O-6-alkylguanine-DNA alkyltransferase	**agt**	CG1303			+		2.1	2.9	1.7	1.7	7.4
pyruvate dehydrogenase kinase	**Pdk**	CG8808			-		2	3.4	2.2	1.5	7.7
CG4576	**CG4576**	CG4576					2.2	4	1.6	2.5	8.5
CG9510 /// CG9515	**CG9510 /// CG9515**	CG33085					2.1	-1.6	-2	1.3	7.4
CG14935 /// ---	**CG14935 /// DsecCG14935**	CG14935					2	2.4	1.2	1.9	7.6
**UNKNOWN**												
**Unknown**												
CG14419	**CG14419**	CG14419	R				10.7	-1.3	-2.1	1.5	8.6
CG13335	**CG13335**	CG13335	R		+		6.2	5.5	3.2	1.7	6.1
CG8160	**CG8160**	CG8160					4.6	2.9	-3.2	9.1	5.5
CG9400	**CG9400**	CG9400					3.6	2.4	-1.9	4.6	9
CG16743	**CG16743**	CG16743		+			3.5	1.7	-1.6	2.7	7.7
CG13077	**CG13077**	CG13077	R	+	+	+	3	-1.1	-1.1	1	4.8
CG7330	**CG7330**	CG7330					3	2.2	-1.6	3.4	7.6
CG34035	**CG34035**	CG34035	R				2.7	-6.6	-2	-3.3	9
CG5953	**CG5953**	CG5953			+		2.6	4.6	1.3	3.5	6.1
CG14417	**CG14417**	CG14417	R				2.6	-4.4	-3.7	-1.2	6.8
CG42319	**CG42319**	CG17041					2.5	2.6	-1.5	3.8	8.9
CG10562	**CG10562**	CG10562					2.5	1.8	1.1	1.6	5.2
CG14515	**CG14515**	CG14515					2.4	-1.2	-2.7	2.3	7.6
CG32040	**CG32040**	CG32040					2.3	3	1.8	1.7	5.2
CG15545	**CG15545**	CG15545					2.3	4.8	2.8	1.7	6
CG10383	**CG10383**	CG10383					2.3	3.8	1.5	2.5	7.7
CG15394	**CG15394**	CG15394			+		2.3	-1.6	-3	1.8	7.1
elastin-like	**Ela**	CG7021					2.1	1.3	1	1.2	8.3
CG14949	**CG14949**	CG14949			+		2.1	5.8	2.5	2.3	5.8
CG31102	**CG31102**	CG31102			-		2.1	1.5	-1.6	2.4	7.9
CG6834	**CG6834**	CG6834					2.1	1.9	-1.2	2.2	6.7
CG5295	**bmm**	CG5295					2	2.6	1.2	2.2	6.7
CG5928	**CG5928**	CG5928					2	2	1.4	1.5	8.9
CG31839	**nimB2**	CG31839					2	2.4	1.5	1.7	9.7
CG9766 /// ---	**CG9766 /// DsimCG9766**	CG9766					2	-1.7	-1.9	1.1	4.2
**Unknown Peptides (<134)**												
Immune induced molecule 4	**IM4**	CG15231		-			12.2	10	1.4	7.1	5.5
CG32185	**CG32185**	CG32185	R		+		12.1	1.1	-1	1.1	5
CG9080	**CG9080**	CG9080	R	+	+	+	8.2	-7.4	-6.5	-1.1	7.1
CG32368	**CG32368**	CG32368	R		+		4.9	31.8	36.7	-1.2	5.1
CG13482	**CG13482**	CG13482	R		+		4.4	1.2	1	1.1	4.5
CG16926	**CG16926**	CG16926					4.2	1	-2.5	2.5	7.3
CG13841 /// CG4000	**CG13841 /// CG4000**	CG13841					2.4	8.5	3.2	2.7	4.9
CG17738	**CG17738**	CG17738			+		2.3	1.3	1	1.3	11.5
CG7294	**CG7294**	CG7294					2.2	2.2	1.1	1.9	10.2
CG16704	**CG16704**	CG16704					2	-1	-2.7	2.7	8.7
**REPRESSED**	**105**	repressed	53	5	8	4					
**STRESS RESPONSE/DETOXIFICATION/ROS**												
**Stress-detoxification**												
Glutathione S transferase D7	**GstD7**	CG4371			+		-5.4	-11.6	-4.3	-2.7	10
Glutathione S transferase D6	**GstD6**	CG4423					-4.2	-7.6	-3	-2.5	7.4
Glutathione S transferase D4	**GstD4**	CG11512			+		-2.2	-2.6	-1.7	-1.6	5
Cyp4s3	**Cyp4s3**	CG9081	R				-2	-2	-2.3	1.2	5.6
**Oxydoreduction**												
Sorbitol dehydrogenase like	**Sodh-1**	CG1982	R	-	-	-	-2.2	-1.8	-1.7	-1	6.5
AOP2-related /// peroxiredoxin 2540	**CG12896 /// Prx2540-1 /// -2**	CG11765					-2.1	-3.4	-2	-1.7	8.2
CG2065	**CG2065**	CG2065			+		-2	-1.6	-1.2	-1.3	9.1
CG3699	**CG3699**	CG3699		-	-	-	-4	-2.8	1.2	-3.2	8.8

CG31559	**CG31559**	CG31559	R				-2.8	-3.5	-5.5	1.6	10.1
**CHITIN/CUTICLE**											
**Chitin binding protein/cuticle**											
chitin-binding peritrophin-A	**Cht5**	CG9307	R				-7.6	-6.5	-3.7	-1.8	10.2
Dm-peritrophin-15b	**Peritrophin-15b**	CG31893					-2.6	-2	-1.3	-1.5	6.4
Acp65Aa	**Acp65Aa**	CG10297					-5	-1.7	5.3	-8.9	9.7
CG1259	**Cpr64Ad**	CG1259	R				-4	-11.3	-6.2	-1.8	12.3
Ecdysone-dependent gene 78E	**Edg78E**	CG7673	R				-2.7	-2.3	-1.8	-1.3	5.2
CG15021	**CG15021**	CG15021			-		-2.3	-3.8	-2.9	-1.3	9
CG32404	**Cpr65Aw**	CG32404					-2.3	-2.2	2.7	-5.7	9.8
Lcp65Af	**Lcp65Af**	CG10533					-2.2	-18.2	-1.8	-9.9	13.2
CG14880	**CG14880**	CG14880	R				-2.1	-1.7	-2.8	1.6	6.9
Lcp65Ad	**Lcp65Ad**	CG6955					-2.1	-2.6	1.7	-4.5	7.3
CG12009	**CG12009**	CG12009					-2.6	-2.1	-1.2	-1.7	7.4
cuticle cluster 8 /// cuticle cluster 7	**Ccp84Aa /// Ccp84Ab**	CG2360					-4.2	-9	-1.5	-5.8	11.7
CG14643	**TwdlG**	CG14643					-2.4	-5.3	-3	-1.8	9.3
GCR(ich)	**TwdlT**	CG5812					-2.8	-3	-1.1	-2.8	9.6
CG5468	**TwdlM**	CG5468	R				-3.2	-4.1	-2.8	-1.4	8
CG6452	**TwdlO**	CG6452	R				-4.4	-13.9	-9.5	-1.5	11.7
CG14534	**TwdlE**	CG14534	R				-5.1	-9.6	-8.2	-1.2	7
CG14240	**TwdlP**	CG14240					-5.8	-2	1.5	-3	10.9
CG5476	**TwdlN**	CG5476	R				-7.9	-7	-2.1	-3.3	10.4
**PEPTIDASE&INHIBITOR**											
**Serine protease**											
CG5255	**CG5255**	CG5255	R				-3.4	-3.5	-2.7	-1.3	5.9
CG31728	**CG31728**	CG31728					-2.7	-2.5	-1.8	-1.4	9.7
CG32808	**CG32808**	CG32808	R				-2.1	-1.4	-1.4	-1	7.7
CG31954	**CG31954**	CG31954					-2	-2	-1.7	-1.2	6.7
**serine protease inhibitor**											
Serine protease inhibitor 1	**Spn43Aa**	CG12172	R				-2.5	-2.1	-1.9	-1.1	5.3
CG1342	**CG1342**	CG1342					-2.6	-3.7	-2.1	-1.7	6.5
Serine protease inhibitor 1	**Spn43Aa**	CG12172	R				-2.5	-2.1	-1.9	-1.1	5.3
CG3604	**CG3604**	CG3604	R	+	+		-3.1	-3.7	-4.2	1.1	11.6
**Other peptidase**											
CG3502	**CG3502**	CG3502	R				-4.6	-7	-3.7	-1.9	8.7
CG8560 /// ---	**CG8560 /// DsecCG8560**	CG8560			-		-3.4	-4.2	-1.4	-3	9.2
CG8773	**CG8773**	CG8773			-		-2	1.1	1.2	-1.1	5.4
CG10280	**CG10280**	CG10280					-2.7	-1.5	1	-1.5	5.9
CG4017	**CG4017**	CG4017	R				-2.7	-3	-2.6	-1.2	6.4
**METABOLISM/TRANSPORTERS**											
**Metabolism**											
CG7529	**CG7529**	CG7529	R				-2.7	-1.4	-1.6	1.2	6.3
CG12480	**CG12480**	CG12480	R				-2.2	-5.3	-6.1	1.2	8.5
CG3940	**CG3940**	CG3940		-	+		-2.2	-3.2	-1.3	-2.4	10.5
SP1029	**SP1029**	CG11956					-2.1	-1	1.2	-1.2	10.2
CG8112	**CG8112**	CG8112					-2	-2.5	-2.5	-1	10.5
**MISCELLANEOUS**											
**Neuronal acitivity-neuropeptide**											
Neuropeptide like precursor protein 1	**Nplp1**	CG3441	R				-2.5	-4.9	-4.3	-1.1	6.1
Odorant-binding protein 99d	**Obp99d**	CG15505	R				-2.4	-3	-10.3	3.4	9.8
syntaxin	**Syx4**	CG2715	R				-2.2	-3.4	-3.8	1.1	6.8
Ptc-related Disp-like	**Ptr**	CG11212	R				-2.1	-1.6	-1.6	1	8.6
**Metal ion binding**											
metallothionein N	**MtnA**	CG9470	R		-		-4.3	1.9	3.6	-1.9	8.9
Metallothionein B	**MtnB**	CG4312					-2.2	-2.2	-1.7	-1.3	4.8
**Various**											
CG6164	**Npc2f**	CG6164		-	-	-	-2	-2.8	-1.9	-1.4	5.5
GV1	**GV1**	CG12023					-2.2	-1.6	1.1	-1.7	11.3
windbeutel	**wbl**	CG7225					-2.4	-2	-1.1	-1.8	9.5
**UNKNOWN**											
**Unknown**											
CG13679	**CG13679**	CG13679	R				-23.7	-116	-28	-4.1	12.6
CG14568	**CG14568**	CG14568	R				-15	-26.4	-12.4	-2.1	9.7
CG14569	**CG14569**	CG14569	R				-12	-38.8	-14.9	-2.6	10.5
CG14573	**CG14573**	CG14573	R				-7.2	-8	-3.6	-2.2	9.1
Osiris	**Osi19**	CG15189	R				-5.8	-10.7	-4.4	-2.5	8.7
CG11345	**CG11345**	CG11345	R				-5.6	-5.6	-3.2	-1.8	11.1
CG14457	**CG14457**	CG14457	R				-5.5	-2.8	-1.4	-1.9	7.4
CG14564	**CG14564**	CG14564	R				-5.3	-7.3	-6.3	-1.2	10.4
CG6908	**CG6908**	CG6908	R	-	-	-	-4.7	-6.3	-6	-1	8.2
CG13059	**CG13059**	CG13059	R				-4.5	-12.2	-6.7	-1.8	11.2
CG13047	**CG13047**	CG13047					-4.4	-6.5	-2.2	-2.9	13.2
CG14565	**CG14565**	CG14565	R				-4.3	-7.8	-9.6	1.2	9.8
CG14457	**CG14457**	CG14457	R				-3.8	-2.3	-1.4	-1.7	6.5
CG11382	**CG11382**	CG11382	R				-3.6	-5.1	-3.9	-1.3	7.7
CG15226	**CG15226**	CG15226					-3.6	-5	1.9	-9.4	8.2
CG11413	**CG11413**	CG11413		+			-3.3	-17.5	-5.9	-3	11.5
CG13239	**CG13239**	CG13239	R				-3.3	-5.8	-4.3	-1.3	8
CG13082	**CG13082**	CG13082	R				-2.9	-2.7	-2.5	-1.1	9.3
CG30458	**CG30458**	CG30458					-2.9	-3	-1.5	-2	9.7

CG14566	**CG14566**	CG14566			-2.8	-4.4	-1.7	-2.6	11.3
CG31606	**CG31606**	CG31606	R		-2.7	-2.6	-2.1	-1.2	5.1
CG14456	**CG14456**	CG14456			-2.6	-4.2	-2.6	-1.6	10.7
CG13046	**CG13046**	CG13046			-2.4	-1.6	-1.2	-1.4	10.2
CG1499	**CG1499**	CG1499			-2.4	1.6	3.9	-2.5	5.9
CG2150	**CG2150**	CG2150	R		-2.4	-3	-2.7	-1.1	6.2
CG15225	**CG15225**	CG15225			-2.2	-2.1	3.8	-8.1	7.1
CG11585	**CG11585**	CG11585			-2	-6.4	-4.2	-1.5	11.9
CG32512	**CG32512**	CG32512			-2.1	-2.4	-1.4	-1.7	7.6
SCP-containing protein A /// B /// C	**scpr-A /// scpr-B /// scpr-C**	CG5207			-2.1	-2.1	-1.1	-1.9	5.9
**Unknown small peptide**									
CG13041	**CG13041**	CG13041			-11.2	-38.1	-3.7	-10.3	9.5
CG13066	**CG13066**	CG13066	R		-9.1	-16	-7.7	-2.1	12.1
CG14324	**CG14324**	CG14324	R		-9.1	-8.7	-7	-1.3	7.2
CG17290	**CG17290**	CG17290	R		-7.2	-15.2	-7.2	-2.1	10.7
CG13060	**CG13060**	CG13060	R		-6.6	-24	-8	-3	8.3
825-Oak /// CG32213	**825-Oak /// CG32213**	CG32214	R		-6.4	-5.6	-3.2	-1.7	8.4
CG30334	**CG30334**	CG30334	R		-6.3	-40.5	-35.7	-1.1	10.7
CG13394 /// GA12253	**CG13394 /// DpseGA25454**	CG13394	R		-6.1	-6.9	-6.5	-1.1	7.4
CG13227	**CG13227**	CG13227	R		-5.6	-6	-2.7	-2.2	9
CG15212	**CG15212**	CG15212	R		-5.5	-8.6	-6	-1.4	8.3
CG34280	**CG34281**	CG14328	R		-4.7	-19.7	-8.9	-2.2	10.9
CG13069	**CG13069**	CG13069			-4.6	-21.1	-5.3	-4	13.7
CG8012	**CG8012**	CG8012			-4.6	-1.1	2.1	-2.4	11.4
CG14096	**CG14096**	CG14096	R		-4.6	-6.8	-3.8	-1.8	9.3
CG15213	**CG15213**	CG15213			-2.7	-12.3	-7.3	-1.7	11.1
CG2444	**CG2444**	CG2444			-2.6	-2.7	-1.9	-1.4	7.2
CG34248	**CG34248**	CG34248			-2.6	-3.8	-2.7	-1.4	9.8
CG34267	**CG34267**	CG34267			-2.5	-7.1	-4.8	-1.5	10.9
CG12546 /// CG14452	**CG12546 /// CG14452**	CG14452			-2.4	-2.4	-1.8	-1.3	5.1
CG18628	**CG18628**	CG18628			-2.2	-2	-1.7	-1.2	4.7
CG32266	**CG32266**	CG32266			-2	-2.2	-2.1	-1.1	13

color legend	<-2	<-6	<-10		
	>2	>4	>6	>8	>10
