# Supplementary figures and images for: Functional Analysis of PGRP-LA in Drosophila Immunity
Source: PLoS One. 2013 Jul 26;8(7):e69742. doi: 10.1371/journal.pone.0069742 (PMC3724876; doi:10.1371/journal.pone.0069742)

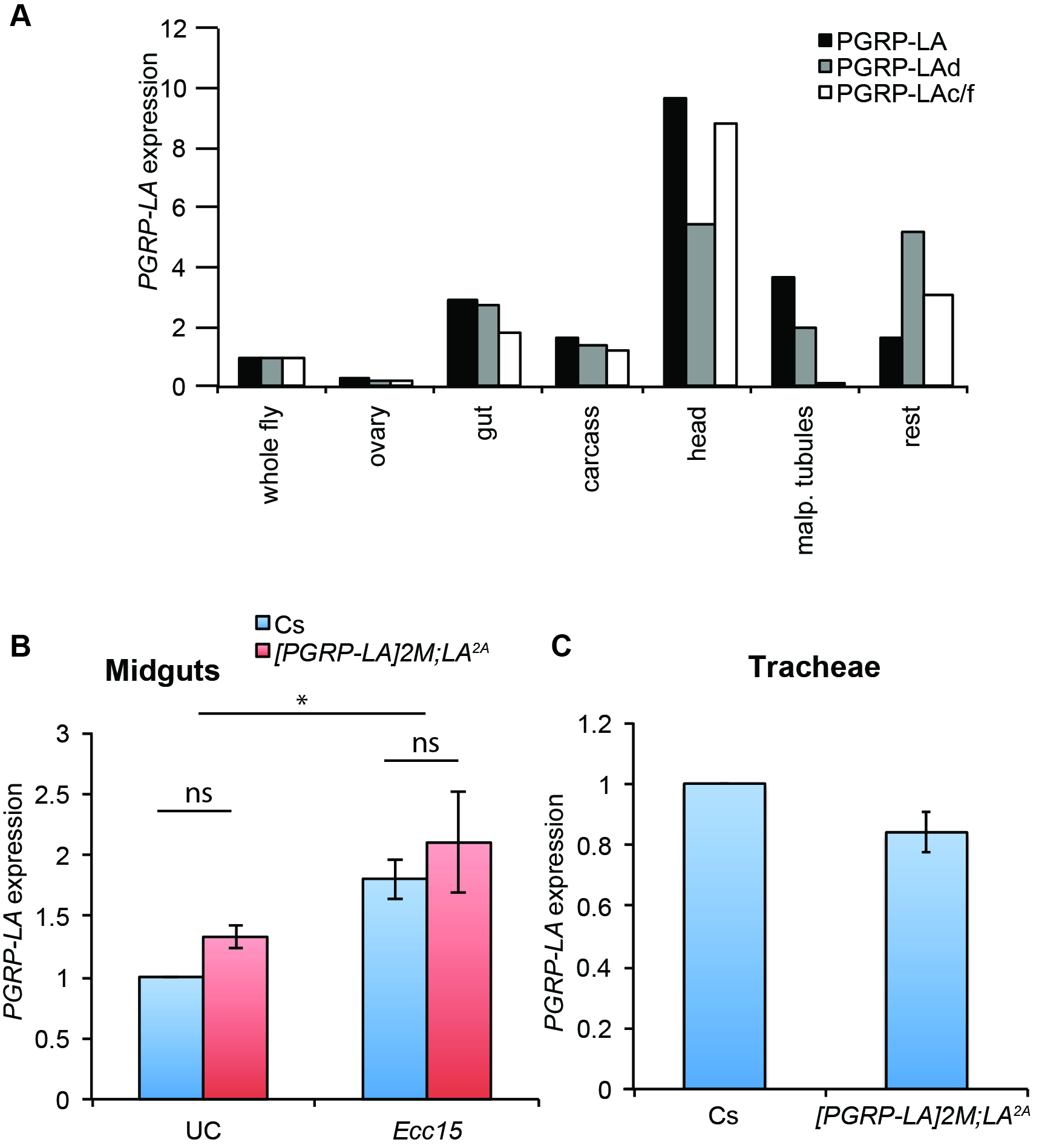

Supplement: Figure S1 — PGRP-LA expression in tissues. RT-qPCR analysis of PGRP-LA expression in wild-type adult female tissues (A) and in adult female midguts (B) and larval tracheae (C) of wild-type and [PGRP-LA]2M; PGRP-LA2A strains. Data are normalized to RpL32 and shown as a ratio of the expression in the wild-type. In A, a single experiment was performed. In B, data are the mean of three independent experiments, error bars indicate standard errors and data were analyzed by 2-way ANOVA with Bonferroni post-tests. In C, data are the mean of two independent experiments and error bars indicate data variation. (TIF) [file pone.0069742.s002.tif]

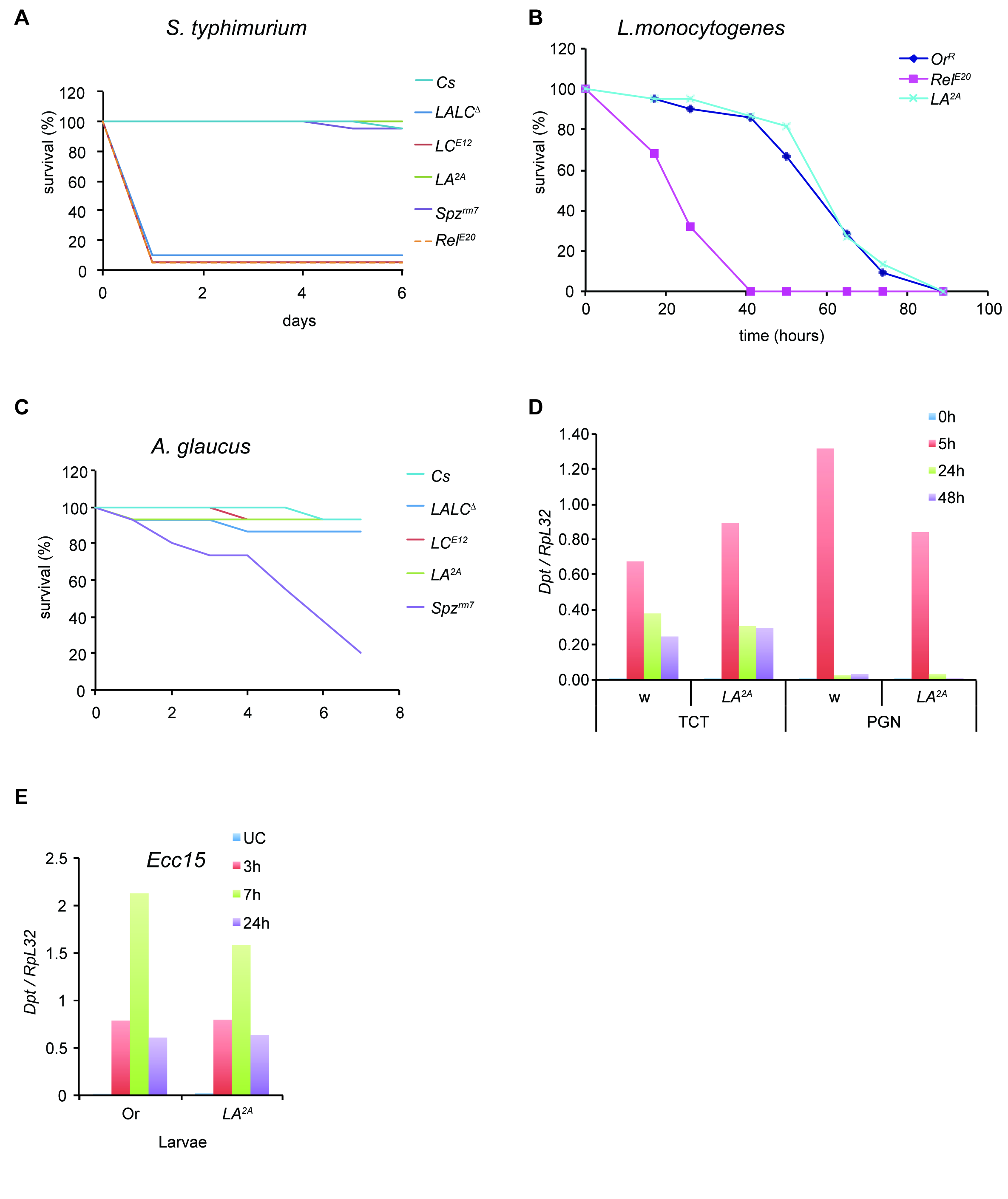

Supplement: Figure S2 — PGRP-LA is not required for the systemic immune response. A–C. Survival analysis of flies after injection with S. typhimurium (A, OD 10−5, 69 nL injected), L. monocytogenes (B, OD 6.5, 9.2 nL injected), A. glaucus (C, spore suspension, 69 nL injected). D,E. Dpt expression after injection of 9.2 nL of monomeric (tracheal cytotoxin, TCT, 0.46 mM) or polymeric peptidoglycan (PGN, 5 mg.mL−1) (A) and after septic injury with Ecc15 in larvae (B). (TIF) [file pone.0069742.s003.tif]

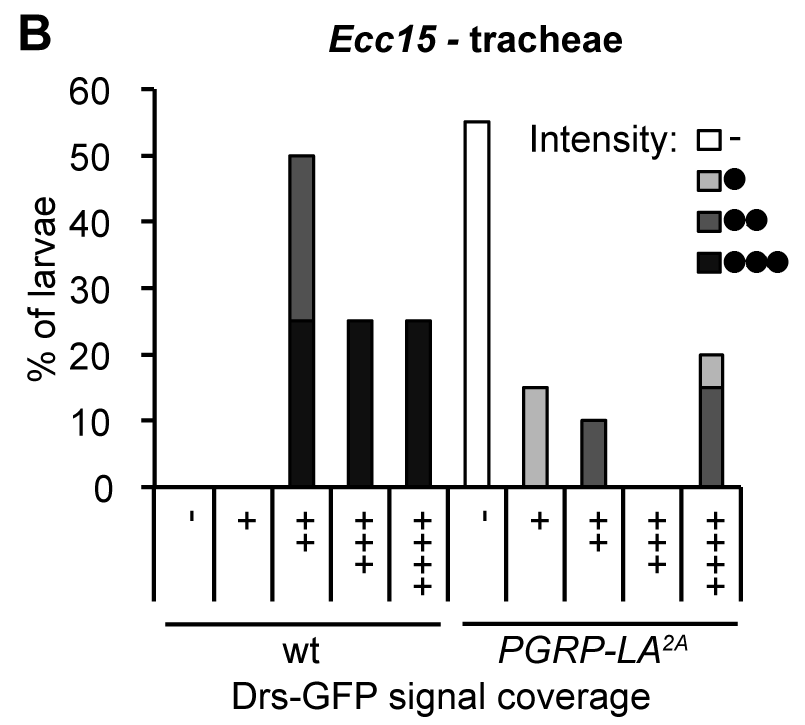

Supplement: Figure S3 — Tracheal Drs response in wt and PGRP-LA2A deficient larvae (GFP). Fluorescence observed in the tracheae of wild-type and PGRP-LA2A larvae expressing the Drs-GFP reporter gene 4 days after bacterial infection with Ecc15 at 18°C. (−) no fluorescence (+) fluorescence in the spiracles only, (++) in the tracheal trunks, (+++) in the tracheae in less than half of the larva or (++++) in the tracheae in more than half of the larva. •, ••, •••: increasing intensity of fluorescence. Data of one experiment representative of 3 independent experiments are shown. (TIF) [file pone.0069742.s004.tif]

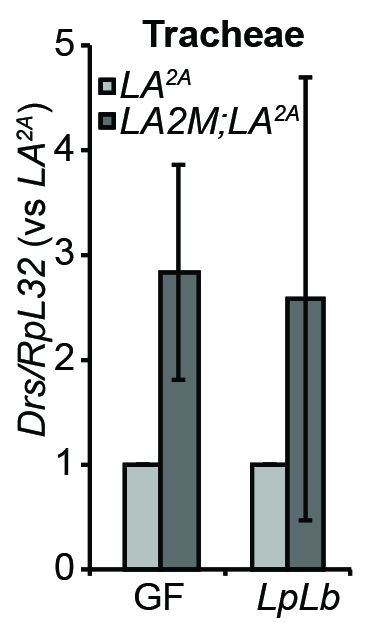

Supplement: Figure S4 — Tracheal Drs response in wt and PGRP-LA2A deficient larvae (RT-qPCR). RT-qPCR quantification of Drs expression in the larval tracheae 24 h after Ecc15 infection in LA2A and [PGRP-LA]2M;LA2A lines raised in germ-free conditions or gnotobiotic conditions (LpLb) where the flora is composed of L. plantarum and L. brevis. Data show the mean of 4 repeats and error bars indicate standard errors. Data were analyzed by Mann-Whitney tests, differences are non significant. (TIF) [file pone.0069742.s005.tif]

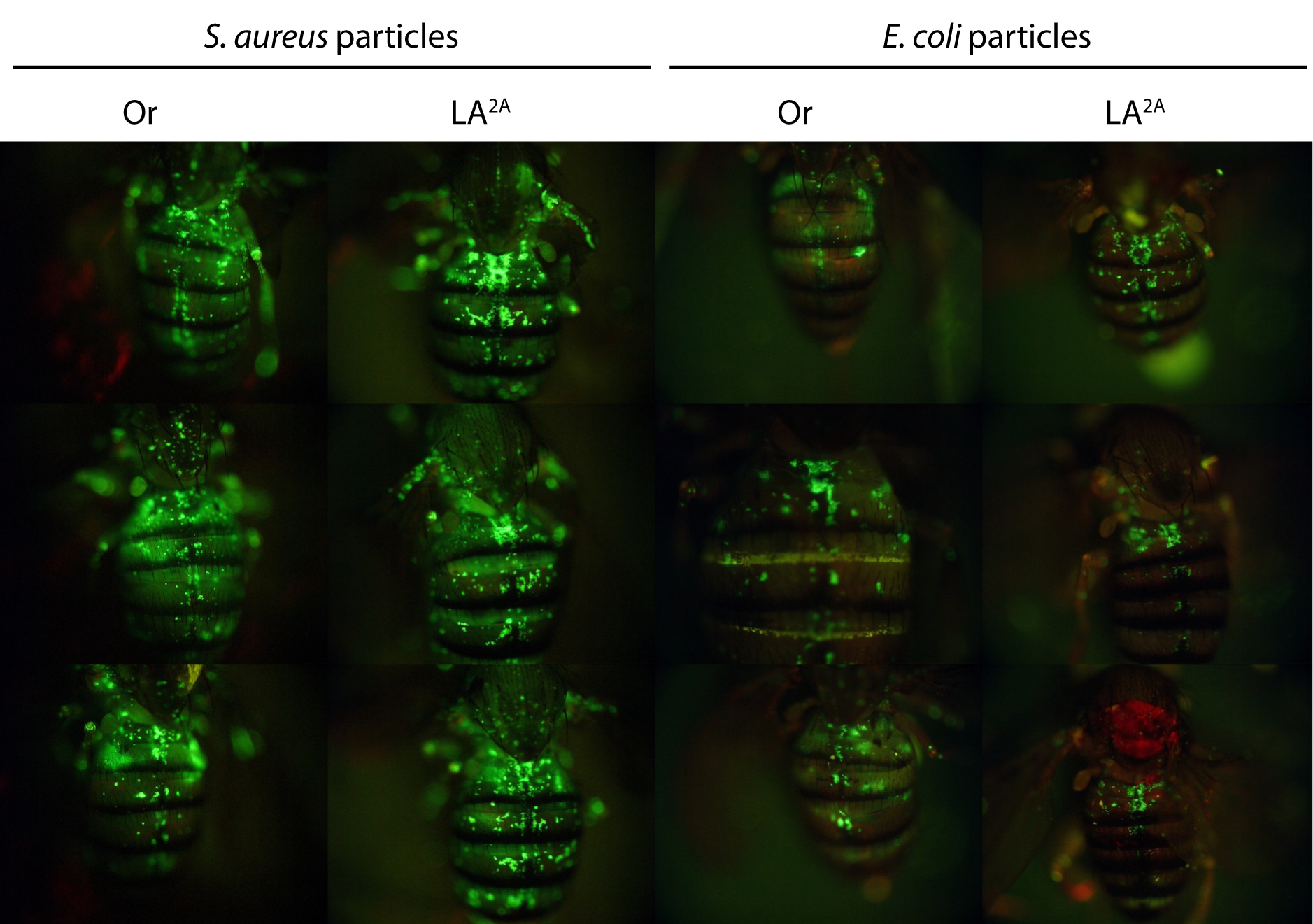

Supplement: Figure S5 — PGRP-LA is not required for the phagocytosis. Fluorescent images of fly abdomens after injection of S. aureus or E. coli nanoparticles. Data show representative results of one experiment. (TIF) [file pone.0069742.s006.tif]
